# Exercise alters brain activation in Gulf War Illness and Myalgic Encephalomyelitis/Chronic Fatigue Syndrome

**DOI:** 10.1093/braincomms/fcaa070

**Published:** 2020-08-10

**Authors:** Stuart D Washington, Rakib U Rayhan, Richard Garner, Destie Provenzano, Kristina Zajur, Florencia Martinez Addiego, John W VanMeter, James N Baraniuk

**Affiliations:** f1 Department of Medicine, Georgetown University Medical Center, 3900 Reservoir Rd., NW Washington, DC 20057, USA; f2 Department of Physiology and Biophysics, Howard University College of Medicine, Adams Building Rm 2420, 520 W Street NW, Washington, DC 20059, USA; f3 Center for Functional and Molecular Imaging, Georgetown University Medical Center, 3900 Reservoir Rd., NW Washington, DC 20057, USA

**Keywords:** post-exertional malaise, verbal working memory, midbrain, Rolandic operculum, cerebellum

## Abstract

Gulf War Illness affects 25–30% of American veterans deployed to the 1990–91 Persian Gulf War and is characterized by cognitive post-exertional malaise following physical effort. Gulf War Illness remains controversial since cognitive post-exertional malaise is also present in the more common Myalgic Encephalomyelitis/Chronic Fatigue Syndrome. An objective dissociation between neural substrates for cognitive post-exertional malaise in Gulf War Illness and Myalgic Encephalomyelitis/Chronic Fatigue Syndrome would represent a biological basis for diagnostically distinguishing these two illnesses. Here, we used functional magnetic resonance imaging to measure neural activity in healthy controls and patients with Gulf War Illness and Myalgic Encephalomyelitis/Chronic Fatigue Syndrome during an N-back working memory task both before and after exercise. Whole brain activation during working memory (2-Back > 0-Back) was equal between groups prior to exercise. Exercise had no effect on neural activity in healthy controls yet caused deactivation within dorsal midbrain and cerebellar vermis in Gulf War Illness relative to Myalgic Encephalomyelitis/Chronic Fatigue Syndrome patients. Further, exercise caused increased activation among Myalgic Encephalomyelitis/Chronic Fatigue Syndrome patients within the dorsal midbrain, left operculo-insular cortex (Rolandic operculum) and right middle insula. These regions-of-interest underlie threat assessment, pain, interoception, negative emotion and vigilant attention. As they only emerge post-exercise, these regional differences likely represent neural substrates of cognitive post-exertional malaise useful for developing distinct diagnostic criteria for Gulf War Illness and Myalgic Encephalomyelitis/Chronic Fatigue Syndrome.

## Teaser

Exercise caused increased blood oxygenation level dependent activation of midbrain, Rolandic operculum and other regions in myalgic encephalomyelitis/chronic fatigue syndrome, but reduced activation of the same regions in Gulf War Illness. These regions may contribute to post-exertional malaise but have opposite pathophysiological mechanisms in the two diseases.

## Graphical Abstract

**Figure:**
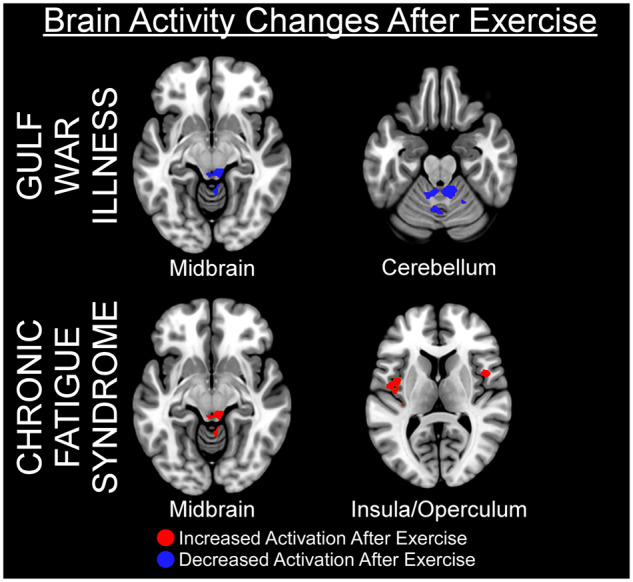

## Introduction

Myalgic Encephalomyelitis/Chronic Fatigue Syndrome (ME/CFS) and Gulf War Illness (GWI) share features of post-exertional malaise (exertional exhaustion), fatigue that is not relieved by rest, unrefreshing and non-restorative sleep, total body pain, systemic hyperalgesia, interoceptive and functional disorders of viscera and disability with severely impaired quality of life (Fukuda *et al.*, 1994; Carruthers *et al.*, 2003; Friedberg *et al.*, 2014). This broad range of symptoms has been difficult to explain by any traditional single medical or psychiatric system of disease.

ME/CFS has been considered to be a chronic consequence following flu-like epidemics (Daugherty *et al.*, 1991) but has a heterogeneous presentation and unknown aetiology. Prevalence is 0.2–2% (Friedberg *et al.*, 2014; Baraniuk, 2019). The 1984 Center for Disease Control (‘Fukuda’) criteria require moderate to severe unremitting fatigue of new onset that persists longer than 6 months and has no explanation despite appropriate medical investigations (Fukuda *et al.*, 1994). At least four of the following eight other criteria must also be present: cognitive complaints of short-term memory or concentration, sore throat, sore lymph nodes, myalgia, arthralgia, headaches including migraine, disordered sleep and post-exertional malaise (i.e. the characteristic, often delayed exacerbation of the entire symptom complex following mild physical, cognitive or emotional efforts). Emphasis has been placed on post-exertional malaise, the characteristic exacerbation of symptoms following minimal effort, as a distinguishing feature of ME/CFS (Carruthers *et al.*, 2003). Biomarker studies suggest roles for cytokines (Hornig *et al.*, 2016), reduced natural killer cell activity (Caligiuri *et al.*, 1987; Mehalick *et al.*, 2018), microRNAs (Baraniuk and Shivapurkar, 2017), metabolomics (Armstrong *et al.*, 2014), gut microbiome (Du Preez *et al.*, 2018), neuroinflammation with microglial activation (Nakatomi *et al.*, 2014) and brainstem connectivity and myelination changes (Barnden *et al.*, 2016, 2018, 2019).

GWI affects ∼25–30% of veterans deployed to the 1990–91 Persian Gulf War. The Centers for Disease Control criteria for Chronic Multisymptom Illness require symptoms from at least two of three symptom clusters: general fatigue, mood and cognitive abnormalities and musculoskeletal pain (Fukuda *et al.*, 1998). An epidemiological comparison of symptoms between deployed and non-deployed Kansas veterans generated criteria requiring three of six domains: fatigue and sleep, pain, neurological/cognitive/mood, gastrointestinal, respiratory and skin symptoms (Steele, 2000). The aetiology has been linked to exposures to neurotoxicants that were present in theatre, including organophosphates, carbamates and other pesticides, sarin/cyclosarin nerve agents and pyridostigmine bromide medication used as prophylaxis against chemical warfare attacks (White *et al.*, 2016). Psychiatric aetiologies have been ruled out.

Differences in proposed aetiology (White *et al.*, 2016) and post-exercise micro-RNA expression in cerebrospinal fluid (Baraniuk and Shivapurkar, 2017) suggest that ME/CFS and GWI are separate disorders. However, symptom overlap between ME/CFS and GWI, notably post-exertional malaise, has promoted unified hypotheses (Mawson and Croft, 2019) and potentially even conflation (Eisen *et al.*, 2005) of both disorders. Further evidence that these are different disorders could be provided by a dissociation between neural substrates underlying post-exertional malaise in ME/CFS and GWI.

Here, we performed functional magnetic resonance imaging (fMRI) on healthy controls (HC) and patients with either ME/CFS or GWI to establish neural substrates of post-exertional malaise. We employed a 2-day submaximal bicycle exercise protocol (Rayhan *et al.*, 2013, 2019) developed to assess the physical and cognitive characteristics of post-exertional malaise (Keech *et al.*, 2015). On the pre- and post-exercise days, cognitive function was assessed by blood oxygen level dependent (BOLD) activity during a continuous version of the N-Back verbal working memory task (Owen *et al.*, 2005; Rottschy *et al.*, 2012). As a first step, whole brain BOLD signal responses were contrasted between groups on the pre-exercise and post-exercise days to assess group-wise differences. Exercise effects within groups were contrasted by paired analysis between days. Standard statistical parametric mapping (SPM12) (Friston *et al.*, 1996) methods identified significant regions of interest (ROIs) with cluster-level *P* < 0.05, family wise error correction (FWE). Activation in these ROIs was compared between groups in exploratory *post hoc* fashion to generate new hypotheses of ME/CFS and GWI neuropathologies (Button, 2019). BOLD signals in each ROI were compared between groups on each day in order to calculate effect sizes (Ellis, 2010) that may be of value for planning future confirmatory studies.

## Materials and methods

### Ethics

All subjects gave written informed consent to this protocol that was approved by the Georgetown University Institutional Review Board (IRB 2009-229, 2013-0943 and 2015-0579) and Department of Defense Congressionally Directed Medical Research Program (CDMRP) Human Research Program Office (HRPO) (A-15547 and A-18479), and listed in clinicaltrials.gov (NCT01291758 and NCT00810225). All clinical investigations were conducted according to the principles expressed in the Declaration of Helsinki.

### Recruitment

GWI, ME/CFS and HC subjects were recruited to this 4-day long in-patient study in the Clinical Research Unit of the Georgetown–Howard Universities Center for Clinical and Translational Science. Subjects had history and physical examinations to ensure their inclusion by meeting Chronic Multisymptom Illness (Fukuda *et al.*, 1998) and Kansas (Steele, 2000) criteria for GWI, Fukuda criteria for ME/CFS (Fukuda *et al.*, 1994), confirmation of sedentary lifestyle for control subjects (less than 40 min of aerobic activity per week) and exclusion because of serious medical or psychiatric conditions such as psychosis (Steele, 2000; Reeves *et al.*, 2003; Jones *et al.*, 2009). History of posttraumatic stress disorder (PTSD) (American Psychiatric Association, 2013) or depression (Spitzer *et al.*, 1999) were not exclusions unless the subject had been hospitalized in the past 5 years. Subjects completed Chronic Fatigue Syndrome Symptom Severity (Baraniuk *et al.*, 2013), SF-36 quality of life (Ware and Sherbourne, 1992), Chalder Fatigue (Cella and Chalder, 2010) and McGill Pain (Dworkin *et al.*, 2009) questionnaires, and had systemic hyperalgesia tested by dolorimetry (Naranch *et al.*, 2002).

### Exercise

Two submaximal bicycle exercise tests were performed 24 h apart. Subjects cycled for 25 min at 70% predicted maximum HR (220 − patient’s age), followed by a climb to 85% maximum HR to reach anaerobic threshold (Garner *et al.*, 2018).

### Verbal working memory task

Subjects practiced the 0-Back and 2-Back working memory task in a mock scanner until they felt proficient. Subjects viewed an instruction panel stating ‘REST’ for 0.8 s followed by 19.2 s of a blank screen. The instruction ‘0-BACK’ was viewed for 0.8 s followed by 1.2 s of a blank screen, and then a string of nine pseudorandomized letters (A, B, C, D) for 0.8 s each followed by 1.2 s of a blank screen per letter. Each time they saw a letter, subjects pressed the corresponding button on an MRI-compatible fibre-optic four button box that was used with both hands. After a second ‘REST’ period, they saw the instruction ‘2-BACK’ and again viewed a string of nine letters. They had to view and remember the first two letters, then press the button for the first letter when they saw the third letter (i.e. ‘2 back’, 4 s delay). The 2-Back task continued for a total of seven responses. This cycle was repeated five times.

### MRI data acquisition and fMRI preprocessing

All structural and functional MRI data were acquired on a Siemens 3 T Tim Trio scanner located within the Center for Functional and Molecular Imaging at Georgetown University Medical Center equipped using a transmit-receive body coil and a commercial 12-element head coil array as described previously (Rayhan *et al.*, 2013). Parameters for structural 3D T1-weighted magnetization-prepared rapid acquisition with gradient echo (MPRAGE) images were: TE = 2.52 ms, TR = 1900ms, TI = 900ms, flip angle = 9°, FOV = 250mm, 176 slices, slice resolution = 1.0 mm and voxel size 1 × 1 × 1mm. Images were processed in SPM12 (Friston *et al.*, 1996). fMRI data consisted of T2*-weighted gradient-echo planar images (EPIs) acquired during the n-back task. EPI data acquisition parameters were: TR/TE = 2500/30 ms, flip angle = 90°, FoV = 205 mm^2^, matrix size = 64 × 64, number of slices = 47, voxel size = 3.2 mm^3^ (isotropic). Raw EPI data were preprocessed through the default pipeline within the CONN toolbox (Whitefield-Gabrieli and Nieto-Castanon, 2012). Briefly, steps were: (i) slice-timing correction, (ii) subject motion estimation and correction, (iii) outlier detection for ‘scrubbing’ based on Artifact Detection Tools, (iv) co-registration with structural data, (v) segmentation and spatial normalization into standard Montreal Neurological Institute (MNI) space (Evans *et al.*, 1993) and (vi) spatial smoothing with a stationary Gaussian filter of 6 mm full-width at half maximum (FWHM). Voxel size was 2.0 mm^3^ (isotropic) after spatial normalization and conversion to Montreal Neurological Institute (MNI) space.

### fMRI data analysis

All within-subject and group-level image analyses were performed in SPM12. The first six scans were removed to account for magnetic saturation. For the first-level analysis, the preprocessed EPI data from each individual were analysed with regressors for instruction, fixation, 0-back, and 2-back as well as estimates of the translation (x, y and z) and rotation (roll, pitch and yaw) as covariates of non-interest. One sample t-tests contrasted the BOLD signals from the 2-back and 0-back. The resulting 2-Back > 0-Back contrast maps (2 > 0-back condition) from every subject were entered into a second-level analysis to compare HC, GWI and ME/CFS groups and the pre- and post-exercise days.

### Statistical analysis

ROIs were determined from the 2-Back > 0-Back contrast maps using a stepped approach in SPM12. The objective was to discover significant candidate voxel clusters (*P* ≤ 0.05, FWE corr) with extent thresholds ≥ 50 voxels that significantly distinguished between groups. Age and gender were included in each model. First, HC, GWI and ME/CFS data were contrasted by two ANOVAs (F-tests for differences between any of the three groups), one for pre-exercise and one for post-exercise data. Second, pairs of groups were contrasted using two-tailed t-tests. This second step is taken because clusters resulting from F-statistics surpassing the threshold for significance could be used to demonstrate that differences existed between the three groups, but ANOVAs can reveal nothing about directions of activation (e.g. HC > GWI or GWI > CFS). Third, exercise effects within groups were found by paired contrasts between the pre- and post-exercise days.

In *post hoc* analysis, ROIs were used as seed regions to better constrain the differences between groups on each day, and the relative exercise-induced alterations for each group. Individual ROIs were assessed by extracting the mean BOLD signal from each subject’s contrast map for each day in the MarsBaR 0.44 toolbox (Brett *et al.*, 2002). MarsBaR outputs for ROIs generated from the voxel-wise ANOVA between the HC, GWI and ME/CFS groups described above were compared by Tukey’s Honest Significant Difference (HSD < 0.05). HSDs by definition could not be reported for the MarsBaR outputs of ROIs yielded from t-tests. *Post hoc* analyses were not performed to identify additional clusters of presumed significance (‘double dipping’) (Kriegeskorte *et al.*, 2009; Vul *et al.*, 2009; Button, 2019) but rather used for hypothesis generation and sample size estimation in this novel data set. Similar methodologies were recently employed to distinguish between autonomic subtypes of GWI using fMRI (Washington *et al.*, 2020).

Effect sizes for whole brain comparisons were estimated for pairs of groups in NeuroPowerTools (Durnez *et al.*, 2016) using Random Field Theory, cluster forming threshold *P* = 0.001, smoothness of 6 mm full width half maximum (FWHM), voxel sizes of 2 × 2 × 2 mm^3^, and 80% power. Outputs were the total sample sizes for future studies and peak lists of voxels that were significantly different across the various contrasts. In addition, the MarsBaR data were used to calculate Hedges’ g (Hedges, 1981) for every pair of groups on the pre-exercise and post-exercise days on the Social Science Statistics website (Stangroom, 2019). Cohen’s d was calculated for within group differences between the pre- and post-exercise scans (Cohen, 1988).

### Visualization of ROIs

The location of each ROI was determined using a custom MATLAB script that employed SPM and xjView 9.6 (http://www.alivelearn.net/xjview/) functions with mapping to the Automated Anatomical Labelling atlas (Tzourio-Mazoyer *et al.*, 2002). Cerebellar regions were confirmed in the SUIT spatially unbiased atlas template (Diedrichsen, 2006; Diedrichsen *et al.*, 2009). Specific nuclei in the midbrain and brainstem were assessed using the Harvard Ascending Arousal Network (AAN) (Edlow *et al.*, 2012) atlas in the Lead DBS software package (Horn and Kuhn, 2015; Horn *et al.*, 2019). Cross-referencing regions defined in different atlases that were based on anatomy, resting state and task related BOLD activation patterns, and functional connectivity was necessary to overcome problems of concordance between atlases created by diverse parcellation schemes (Bohland *et al.*, 2009; Arslan *et al.*, 2018). Each ROI was depicted by sagittal (x-axis), coronal (y-axis) and axial (z-axis) views through the voxel with the highest magnitude BOLD signal.

### Data availability

Data are available from the authors upon request and will be uploaded to OpenfMRI and/or the Boston Biorepository, Recruitment, and Integrative Network (BBRAIN) for GWI.

## Results

### Demographics

Subject demographics are summarized in Table 1. Mean ± 95% CI that were significantly different from the other two groups were determined by ANOVA and Tukey HSD < 0.05 (*) or Fisher Exact Tests (^†^*P* < 0.0001). As anticipated from the demographics of each disease, the ME/CFS group (*N* = 38) had more females than the HC (*N* = 31) and GWI (*N* = 80) groups. Measures of symptoms and disability were more severe in ME/CFS and GWI than HC, and indicated significant pain, tenderness (systemic hyperalgesia by dolorimetry), fatigue and disruption of quality of life. Due to potential gender differences, all SPM contrasts included age and gender as co-variates. PTSD and depression were more prevalent in GWI. PTSD subjects did not have derealization or depersonalization of the dissociative subtype (Harricharan *et al.*, 2016).

**Table 1:** Demographics

	Healthy control	ME/CFS	GWI
*N*	31	38	80
Age	43.2 ± 5.8	47.7 ± 4.1	46.9 ± 1.6
% Male	61.30%	29.0%^†^	77.50%
BMI	28.3 ± 1.6	26.2 ± 1.7	29.6 ± 1.2
McGill pain score	3.3 ± 2.1*	13.5 ± 3.4	23.7 ± 2.0
Dolorimetry (kg)	6.1 ± 0.6*	3.8 ± 0.5	3.6 ± 0.4
Chalder fatigue	12.3 ± 1.9*	23.0 ± 2.0	25.3 ± 1.1
History of PTSD (%)^†^	9.7%	15.8%	45.0%
Major depression by PRIMEMD (%)^†^	0%	18.2%	50.0%
CFS symptom severity			
Fatigue	1.3 ± 1.6*	3.4 ± 0.3	3.5 ± 0.2
Memory and concentration	1.2 ± 0.4*	2.9 ± 0.3	3.1 ± 0.2
Sore throat	0.3 ± 0.1	1.0 ± 0.3	1.4 ± 0.3
Sore lymph nodes	0.1 ± 0.1	1.0 ± 0.3	1.5 ± 0.3
Muscle pain	0.6 ± 0.3*	2.5 ± 0.4	3.1 ± 0.2
Joint pain	0.8 ± 0.4*	1.8 ± 0.5	3.2 ± 0.2
Headaches	1.0 ± 0.4*	2.0 ± 0.4	2.7 ± 0.3
Sleep	1.7 ± 0.5*	3.2 ± 0.3	3.5 ± 0.2
Exertional exhaustion	0.6 ± 0.4*	3.5 ± 0.3	3.4 ± 0.2
SF-36			
Physical functioning	85.2 ± 8.5*	45.9 ± 8.5	46.4 ± 5.3
Role-physical	80.0 ± 13.0*	9.5 ± 8.0	9.4 ± 5.4
Bodily pain	82.9 ± 7.1*	46.6 ± 8.6	29.3 ± 3.9
General health	69.8 ± 8.0*	34.7 ± 7.5	26.3 ± 4.2
Vitality	60.2 ± 7.3*	18.9 ± 5.1	16.8 ± 3.4
Social functioning	80.0 ± 8.8*	32.4 ± 8.7	30.4 ± 5.3
Role-emotional	86.7 ± 10.9	69.4 ± 14.2	30.3 ± 8.4*
Mental health	73.6 ± 5.9	67.7 ± 5.4	54.3 ± 4.9*

Questionnaire scores were significantly different between SC, CFS and GWI by ANOVA followed bys Tukey Honest Significant Difference (*P < 0.05) to correct for multiple comparisons. Self-reported history of PTSD and risk of major depression were higher in GWI (^†^*P* < 0.05 by Fishers Exact Test for 2 × 3 contingency tables).

### fMRI

**Figure 1:**
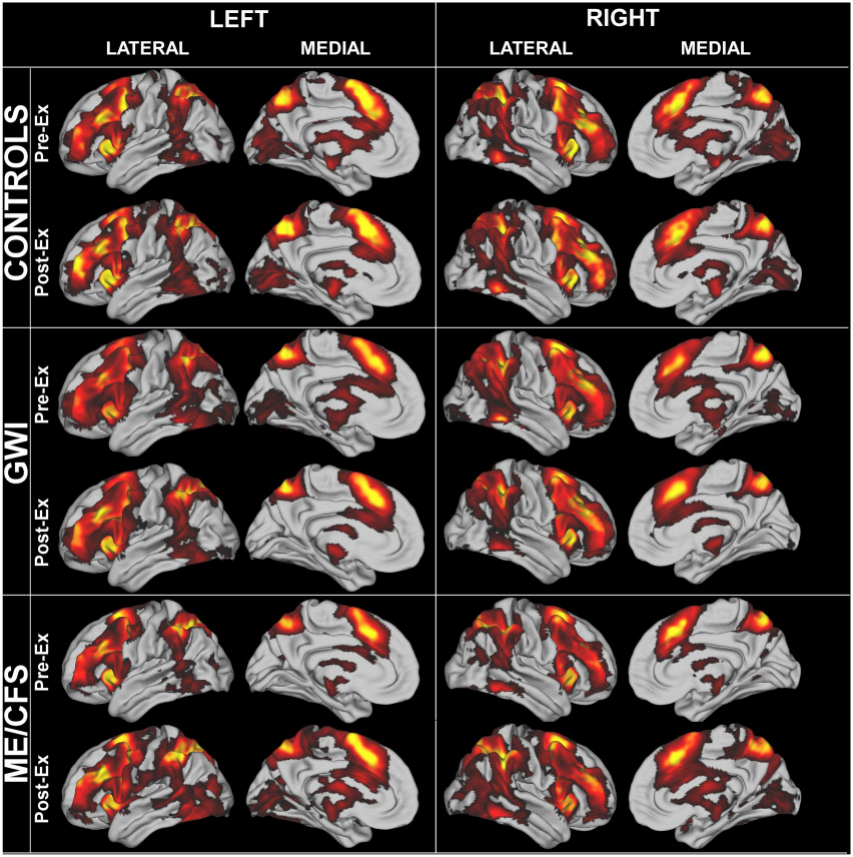
**Cortical BOLD activation for 2-Back > 0-Back.** Frontal parietal executive control network regions were activated in the HC, GWI and ME/CFS groups during the pre-exertion and post-exertion scans.

General patterns of BOLD activation for the 2-Back > 0-Back condition after corrections for age and gender were similar between HC, ME/CFS and GWI groups before and after exercise, and showed activation of frontal parietal executive control regions as anticipated for this working memory task (Fig. 1).

### Pre- versus post-exercise differences in HC, GWI and ME/CFS activity

**Figure 2:**
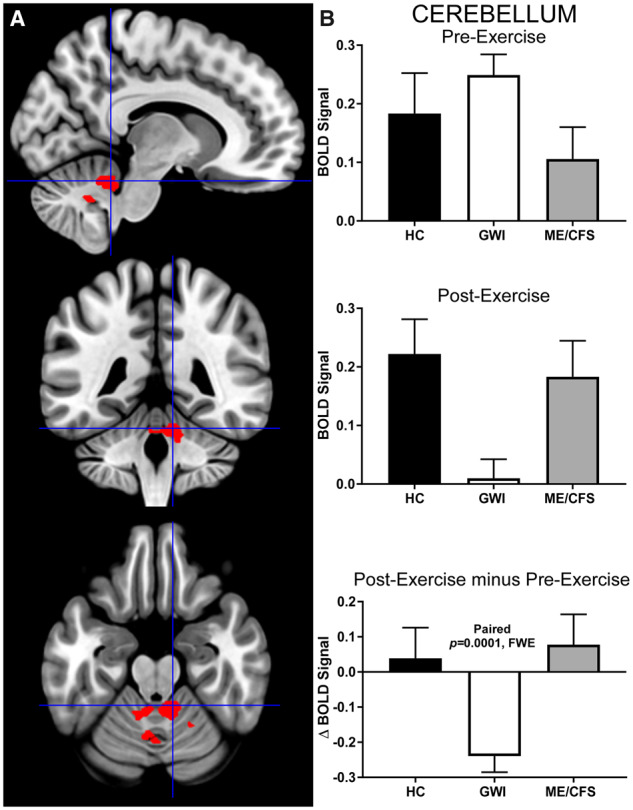
**Cerebellum.** A cerebellar ROI was identified by contrasting pre-exercise > post-exercise activity derived from the 2-Back > 0-Back condition in the GWI group (cluster-level *P* = 0.0028, FWE, k_E_ = 396). (**A**) Sagittal (top), coronal (middle) and transverse (bottom) slices of an MNI-standard brain, where crosshairs indicate the cluster’s most active voxel (10, −44, -22). (**B**) BOLD response for the 2-Back > 0-Back condition (mean ± SEM) are shown for pre-exercise (top) and post-exercise (middle). ΔBOLD (bottom) is the post-minus pre-exercise BOLD response for the 2-Back > 0-Back condition for the control (black bars), GWI (white bars) and ME/CFS (grey bars). Error bars represent 95% CI.

The voxel-wise paired t-tests paired comparisons yielded no significant cluster-level pre- versus post-exertional differences in either the HC or ME/CFS groups. However, the pre-exercise > post-exercise contrast in GWI yielded an ROI (cluster-level: *P* = 0.00013, FWE; k_E_ = 396) located in the cerebellum (Fig. 2). The post-exercise > pre-exercise contrast in GWI yielded no significant clusters. This cerebellar ROI was composed of 66% gray matter (260/396), portions of which included right vermis IX (51/396, 13%), left lobule IX (30/396, 8%), left lobule IV/V (21/396, 5%), and right lobules III (21/396, 5%) and IV/V (20/396, 5%), per the Automated Anatomical Labelling atlas. Exercise decreased activation in this cerebellar ROI only in the GWI group. Before exercise, all three groups had equal levels of activation. The large effect size of this paired comparison (Cohen’s d = 0.79) suggest a high likelihood of replicating this result in future studies.

### Group differences between HC, GWI and ME/CFS

The ANOVA comparing the groups for 2-Back > 0-Back pre-exercise yielded no significant cluster-level group differences. The ANOVA comparing the groups for 2-Back > 0-Back post-exercise yielded three significant clusters plus two trending clusters. Significant clusters included left sensorimotor cortex (cluster level: *P* = 0.004, FWE; k_E_ = 180), left cuneus/precuneus (cluster level: *P* = 0.046, FWE; k_E_ = 109) and midbrain/isthmus (cluster level: *P* = 0.040, FWE; kE = 113). Trending clusters included right intraparietal sulcus (cluster level: *P* = 0.051, FWE; k_E_ = 106) and left Rolandic operculum (cluster level: *P* = 0.066, FWE; k_E_ = 99). ROI Analyses of ROIs resulting from ANOVAs are reported in Supplementary Figs 1–5).

Since voxel-wise ANOVAs provide no information regarding specific group differences that resulted in clusters (e.g. HC>ME/CFS or ME/CFS>GWI) nor the relative directions and magnitudes of these differences, we performed additional voxel-wise *post hoc* comparisons between the HC, GWI and ME/CFS groups. These comparisons generated several ROIs, which form the bases of our results going forward. We will describe these ROIs here based on their voxel counts (k_E_) and regional composition percentages. In subsequent sections, we discuss their statistical significance and contextualize them based on whether exercise caused regional activation to decrease in GWI or increase in ME/CFS.

*Post hoc* voxel-wise two-sample *t*-test comparisons of 2-Back > 0-Back contrasts revealed significant effects of exercise in the HC, GWI and ME/CFS groups. There were no differences between the three groups before exercise except GWI>ME/CFS. Before exercise, GWI>ME/CFS yielded an ROI (k_E_ = 274) located in the right angular gyrus (243/274 voxels, 89%) and inferior parietal lobule (24/274, 9%). After exercise, GWI>ME/CFS yielded no significant clusters, but ME/CFS>GWI yielded four. The first of these four ROIs (k_E_ = 269) was located in the right superior (165/269 voxels, 61%) and inferior (34/269, 13%) parietal lobules, and extended to the precuneus (32/269, 12%) and angular gyrus (18/269, 7%). The second of these (k_E_ = 141) was located in the dorsal midbrain. The third of these ROIs (k_E_ = 489) was located in the right middle insula (95/489, 19%), putamen (95/489, 19%), temporal pole (69/489, 14%), Rolandic operculum (40/489, 8%), superior temporal gyrus (34/489, 7%) and amygdala (24/489, 5%). The last of these four ROIs (k_E_ = 273) was located in the left Rolandic operculum (136/273 voxels, 50%) and posterior insula (131/273, 48%). After exercise, ME/CFS>HC yielded an ROI (k_E_ = 373) in the left Rolandic operculum (192/373 voxels, 51%), posterior insula (103/373, 28%) and superior temporal gyrus (26/373, 7%). Respectively, these ROIs will hereafter be referred to as (i) right angular gyrus, (ii) right intraparietal sulcus, (iii) midbrain, (iv) right middle insula, (v) 273-voxel Rolandic operculum and (vi) 373-voxel Rolandic operculum. With the exception of the sensorimotor cortex ROI, all of the ROIs resulting from the post-exercise ANOVA have close spatial correspondence with these *post hoc* ROIs, and their degree of overlap is described below.

### ROIs with decreased activation after exercise in GWI

Some of the significant, cluster-level differences between the ME/CFS and GWI groups emerge due to post-exercise reductions in BOLD activation in the GWI group. ROIs conforming to this activation pattern are summarized below.

**Figure 3:**
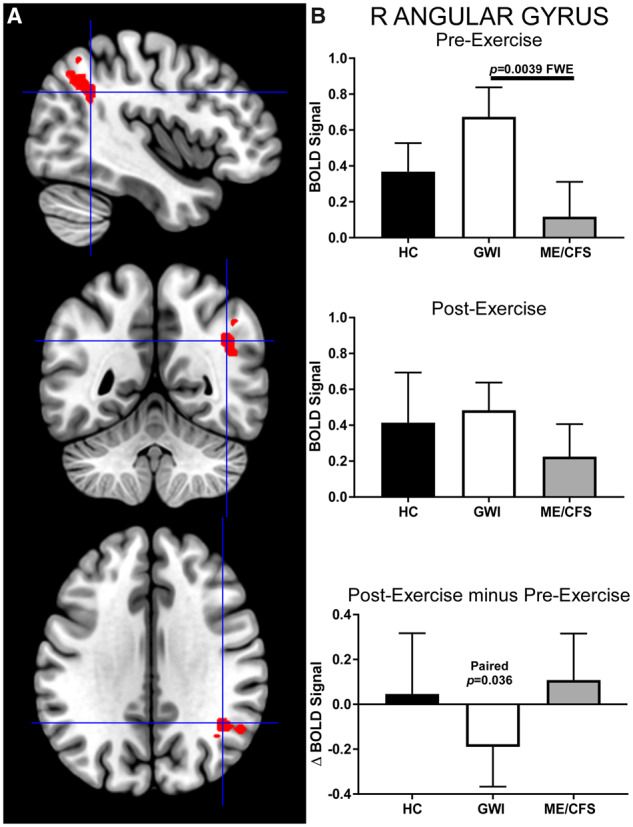
**Right angular gyrus.** This ROI was identified by contrasting GWI>ME/CFS group BOLD activity derived from the 2-Back > 0-Back condition elicited before exercise (cluster-level *P* = 0.0039, FWE; k_E_ = 274). (**A**) Sagittal (top), coronal (middle) and transverse (bottom) slices of an MNI-standard brain, where crosshairs indicate the cluster’s most active voxel (42, −52, 32). (**B**) BOLD response for the 2-Back > 0-Back condition (mean ± SEM) are shown for pre-exercise (top) and post-exercise (middle). ΔBOLD (bottom) is the post-minus pre-exercise BOLD response for the 2-Back > 0-Back condition for the control (black bars), GWI (white bars) and ME/CFS (grey bars). Error bars represent 95% CI.

The right angular gyrus (cluster-level: *P* = 0.0039, FWE; k_E_ = 274) was the only ROI to have significantly different activation between ME/CFS and GWI before exercise (Fig. 3). Specifically, BOLD signal was greater in GWI than ME/CFS before exercise. Exercise decreased the activation in GWI (paired *t*-test: *P* = 0.036) but had no significant effect on ME/CFS. However, effect sizes were small (Hedges’ *g* < 0.40), suggesting that these differences may be difficult to replicate in future studies.

**Figure 4:**
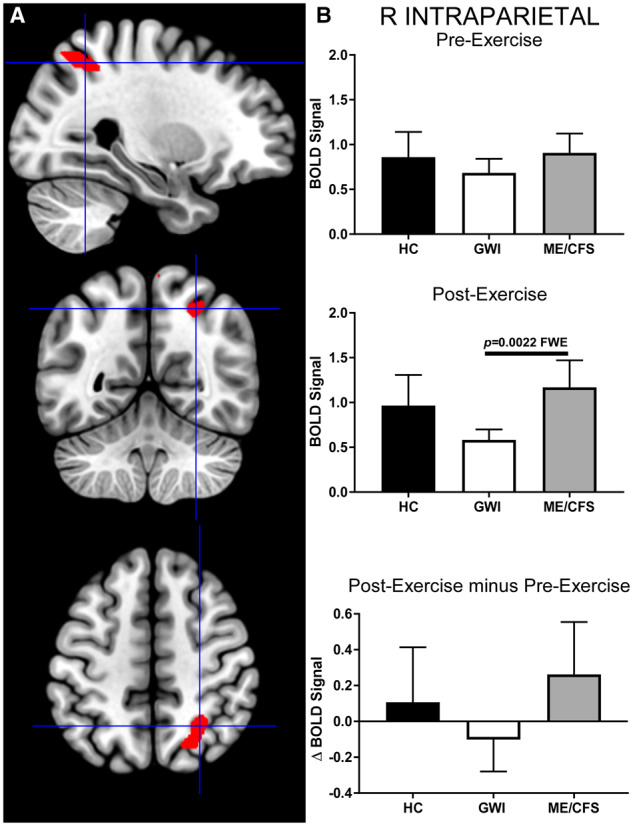
**Right intraparietal sulcus.** This ROI was identified by contrasting ME/CFS>GWI group BOLD activity derived from the 2-Back > 0-Back condition elicited after exercise (cluster-level *P* = 0.0022, FWE; k_E_ = 269). (**A**) Sagittal (top), coronal (middle) and transverse (bottom) slices of an MNI-standard brain, where crosshairs indicate the cluster’s most active voxel (28, −56, 50). (**B**) BOLD response for the 2-Back > 0-Back condition (mean ± SEM) are shown for pre-exercise (top) and post-exercise (middle). ΔBOLD (bottom) is the post-minus pre-exercise BOLD response for the 2-Back > 0-Back condition for the control (black bars), GWI (white bars) and ME/CFS (grey bars). Error bars represent 95% CI.

The GWI group had significantly less activation in the right intraparietal sulcus ROI (cluster-level: *P* = 0.0022, FWE; k_E_ = 269) than the ME/CFS group after exercise (Fig. 4). Neither GWI nor ME/CFS showed significant, pair-wise differences in activation (ΔBOLD) after exercise. Thus, these differences likely emerged due to relatively small post-exertional increases and decreases in ME/CFS and GWI BOLD activity, respectively. However, effect sizes were large (Hedges’ *g *= 0.87), suggesting that these differences are likely to be replicated in future studies. There was a complete overlap between voxels in this ROI and the right intraparietal sulcus ROI resulting from the ANOVA trend, such that all voxels in the latter were contained within the former. Both ROIs overlapped with the precuneus default mode network node as defined by Shirer *et al.* (2012). Neither of the right intraparietal sulcus ROIs overlapped with the right angular gyrus ROI.

**Figure 5:**
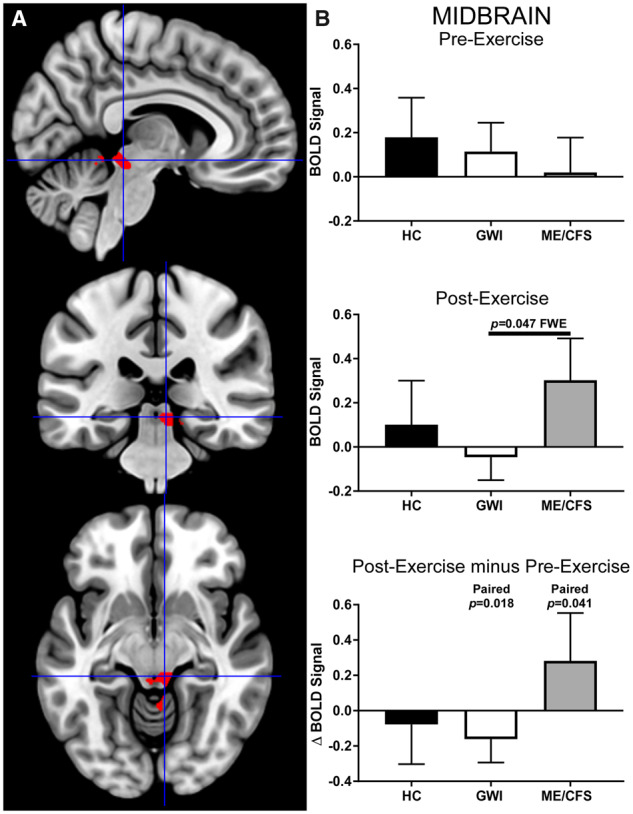
**Midbrain.** The midbrain ROI identified by contrasting ME/CFS>GWI group BOLD activity derived from the 2-Back > 0-Back condition elicited after exercise (cluster-level *P* = 0.047, FWE; k_E_ = 141). (**A**) Sagittal (top), coronal (middle) and transverse (bottom) slices of an MNI-standard brain, where crosshairs indicate the cluster’s most active voxel (6, −32, −10). (**B**) BOLD response for the 2-Back > 0-Back condition (mean ± SEM) are shown for pre-exercise (top) and post-exercise (middle). ΔBOLD (bottom) is the post-minus pre-exercise BOLD response for the 2-Back > 0-Back condition for the control (black bars), GWI (white bars) and ME/CFS (grey bars). Error bars represent 95% CI.

The midbrain ROI (cluster-level: *P* = 0.047, FWE; k_E _= 141) had significantly less activation in the GWI than in the ME/CFS group after exercise (Fig. 5). Exercise caused changes in opposite directions, with deactivation for GWI (paired *t*-test: *P* = 0.018) yet increased activation for ME/CFS (paired *t*-test: *P* = 0.041). The large effect size (Hedges’ *g* = 0.69) suggests that these differences are likely to be replicated in future studies. Per the Harvard Ascending Arousal Atlas, the rostral end of the midbrain ROI extended from the dorsal midbrain in the periaqueductal gray (PAG) (21/141 voxels, 15%), through the adjacent right midbrain reticular formation (11/141, 8%), and caudally to the right pendunculotegmental (formerly pedunculopontine, PPN) nucleus (5/141 voxels, 4%) of the isthmus of the rostral hindbrain. Based on ROIs derived by downsampling from the high-resolution Sitek-Gulban Atlas (Sitek *et al.*, 2019), this midbrain ROI also engulfs 23% (3/13) of left and 43% (6/14) of right inferior colliculus. The ROI did not extend to the ventral midbrain or pontine nuclei.

### ROIs with increased activation after exercise in ME/CFS

Other significant cluster-level differences between the HC, GWI and ME/CFS groups emerge due to post-exercise increases in BOLD activation in the ME/CFS group. In general, these ROIs reside in operculo-insular cortex and are characterized by deactivation for the 2-Back > 0-Back condition across the HC, GWI and ME/CFS groups before exercise. This deactivation persists after exercise for the HC and GWI groups but not the ME/CFS group, which either activates or simply fails to deactivate.

**Figure 6:**
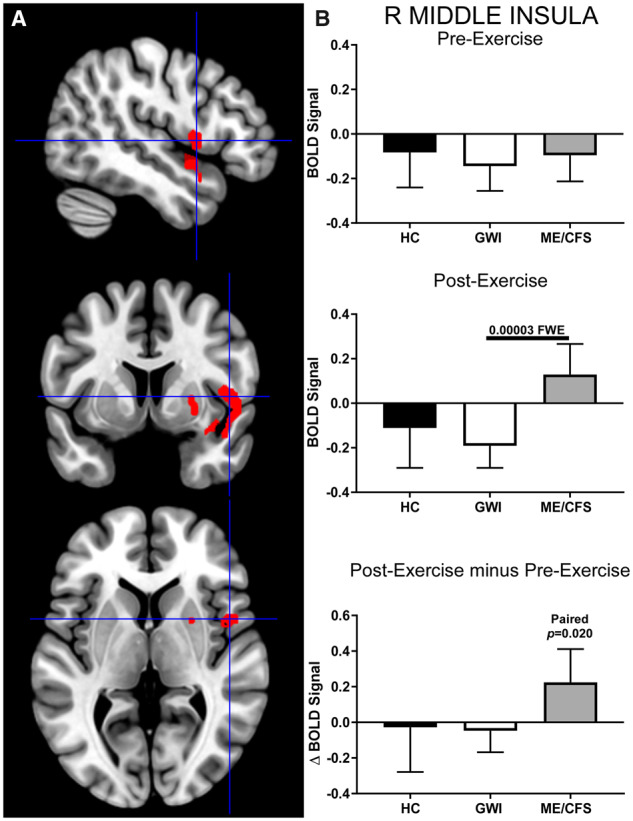
**Right middle insula.** This ROI was identified by contrasting ME/CFS>GWI group BOLD activity derived from the 2-Back > 0-Back condition elicited after exercise (cluster-level *P* = 0.000030, FWE; k_E_ = 489). (**A**) Sagittal (top), coronal (middle), and transverse (bottom) slices of an MNI-standard brain, where crosshairs indicate the cluster’s most active voxel (48, 6, 4). (**B**) BOLD response for the 2-Back > 0-Back condition (mean ± SEM) are shown for pre-exercise (top) and post-exercise (middle). ΔBOLD (bottom) is the post-minus pre-exercise BOLD response for the 2-Back > 0-Back condition for the control (black bars), GWI (white bars) and ME/CFS (grey bars). Error bars represent 95% CI.

The right middle insula ROI (cluster-level: *P* = 0.000030, FWE; k_E_ = 489) was deactivated in the HC, GWI and ME/CFS groups before exercise but had significantly higher activity in the ME/CFS group than in GWI following exercise (Fig. 6). Specifically, exercise caused a significant increase in BOLD signal only for the ME/CFS group (paired *t*-test: *P* = 0.02). Exercise had no effect on GWI or HC. The difference between ME/CFS and GWI in the right middle insula ROI following exercise had a large effect size (Hedges’ *g *= 0.73), suggesting that these differences are likely to be replicated in future studies.

**Figure 7:**
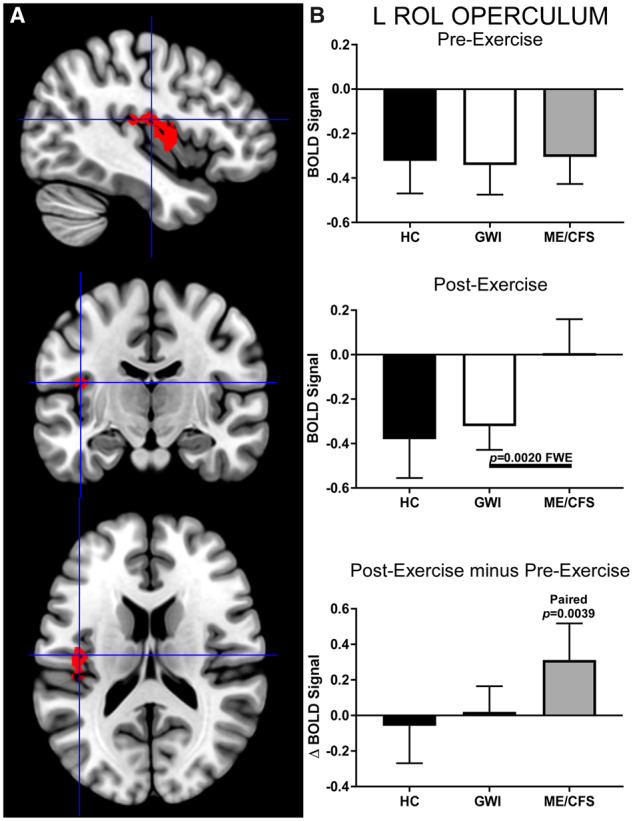
**Left Rolandic operculum (k_E_ = 273).** This ROI was identified by contrasting ME/CFS>GWI group BOLD activity derived from the 2-Back > 0-Back condition elicited after exercise (cluster-level *P* = 0.0020, FWE; k_E_ = 273). (**A**) Sagittal (top), coronal (middle), and transverse (bottom) slices of an MNI-standard brain, where crosshairs indicate the cluster’s most active voxel (−42, −14, 16). (**B**) BOLD response for the 2-Back > 0-Back condition (mean ± SEM) are shown for pre-exercise (top) and post-exercise (middle). ΔBOLD (bottom) is the post- minus pre-exercise BOLD response for the 2-Back > 0-Back condition for the control (black bars), GWI (white bars) and ME/CFS (grey bars). Error bars represent 95% CI.

Similarly, in the left 273-voxel Rolandic operculum ROI (cluster-level: *P* = 0.002, FWE; k_E_ = 273), all three groups were deactivated before exercise but the ME/CFS group had significantly higher activity than GWI after exercise (Fig. 7). Here, exercise caused BOLD signal in the ME/CFS group to remain at baseline levels (paired *t*-test: *P* = 0.0039) while affecting no change on the deactivation patterns observed before exercise in HC or GWI. These differences are likely to be replicated in future studies, due to the large effect size (Hedges’ *g* = 0.69).

**Figure 8:**
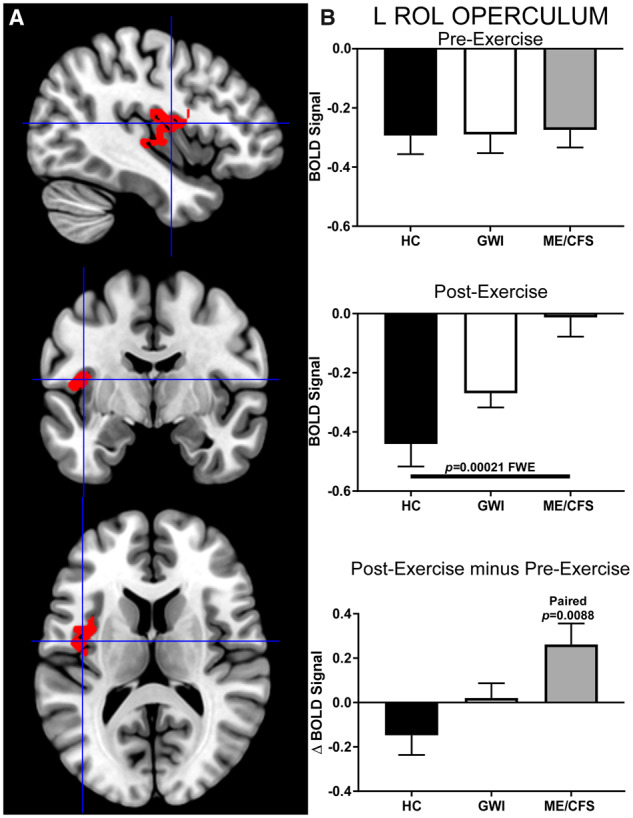
**Left Rolandic operculum (k_E_ = 373).** The larger left Rolandic operculum ROI was identified by contrasting ME/CFS>HC group BOLD activity derived from the 2-Back > 0-Back condition elicited after exercise (cluster-level *P* = 0.00021, FWE; k_E_ = 373). (**A**) Sagittal (top), coronal (middle), and transverse (bottom) slices of an MNI-standard brain, where crosshairs indicate the cluster’s most active voxel (−42, −6, 12). (**B**) BOLD response for the 2-Back > 0-Back condition (mean ± SEM) are shown for pre-exercise (top) and post-exercise (middle). ΔBOLD (bottom) is the post-minus pre-exercise BOLD response for the 2-Back > 0-Back condition for the control (black bars), GWI (white bars) and ME/CFS (grey bars). Error bars represent 95% CI.

This pattern was again observed in the left 373-voxel Rolandic operculum ROI (cluster-level: *P* = 0.00021, FWE; k_E_ = 373). Before exercise, HC, GWI and ME/CFS showed deactivation in this ROI. Exercise caused BOLD signal in the ME/CFS group to remain at baseline (paired *t*-test: *P* = 0.0088), while having no significant effects on the deactivation patterns in HC and GWI in this region (Fig. 8). The effect size was very large (Hedges’ *g* = 1.05), suggesting a high likelihood for replicating this result in future studies.

**Figure 9:**
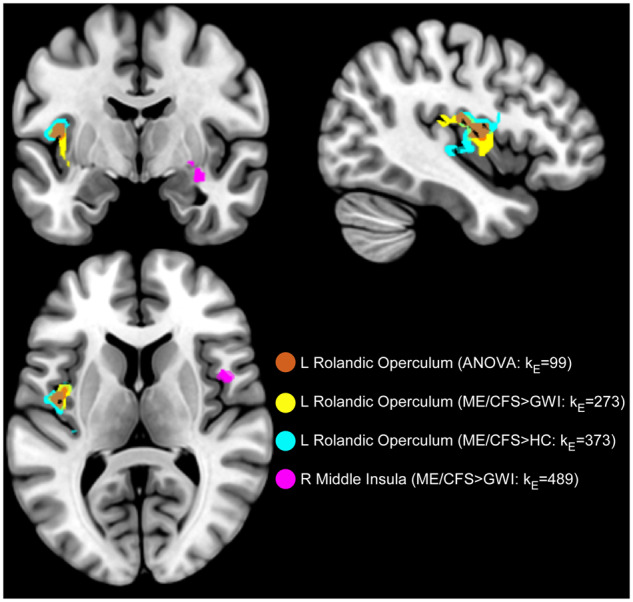
**Overlap of operculo-insular ROIs.** Operculo-insular cortical regions with elevated activity after exercise in ME/CFS. Three overlapping regions of the left Rolandic operculum and right posterior to middle insula were identified via ANOVA between HC, GWI and ME/CFS (red/brown, *P* = 0.066, FWE; k_E_ = 99) as well as *post hoc* contrasts between CFS > HC (cyan; *P* = 0.00021, FWE; k_E_ = 373) and CFS > GWI (yellow; *P* = 0.0020, FWE; k_E_ = 273). The CFS > GWI contrast also revealed a significant right middle insula ROI (magenta, *P* = 0.000030, FWE; k_E_ = 489).

There was an overlap between the 273- and 373-voxel Rolandic operculum ROIs, such that 45% (124/273) of voxels in the former corresponded to 33% (124/373) in the latter. The Rolandic operculum ROI resulting from a trend in the post-exercise ANOVA (k_E_ = 99) overlapped 95% (94/99) and 94% (93/99) with the 273- and 373-voxel Rolandic operculum ROIs (Fig. 9).

## Discussion

The general patterns of bilateral frontal parietal executive control network and dorsal attention network activation during the 2-Back > 0-Back condition were similar pre- and post-exercise, between groups, and conformed to previously described patterns for working memory and N-Back testing (Owen *et al.*, 2005; Rottschy *et al.*, 2012; Caseras *et al.*, 2013).

Working memory involves processes of storage to actively maintain representations in memory and filtering to inhibit irrelevant information from entering storage. In young adults (mean age 25.7 years), storage of information was mediated by the neuronal network of the bilateral posterior parietal cortex, left ventromedial prefrontal cortex and right precuneus (Vellage *et al.*, 2016). Filtering involved regions in the bilateral anterior insulae, right brainstem and right cerebellum. An older group (mean age 65.8 years) augmented the filtering process by recruiting additional bilateral anterior insulae regions, right precuneus and bilateral ventromedial prefrontal cortex. Their storage network was expanded by the recruitment of portions of the bilateral ventral prefrontal cortex, the superior, middle and inferior temporal cortex, left cingulum and bilateral parahippocampal cortex. The ME/CFS and GWI groups did not expand their utilization of these regions during their N-Back testing after exercise suggesting that they did not have significant dysfunction with filtering and storage activities.

The only ROI that was significantly different between groups before exercise was the right angular gyrus, where GWI had a greater activation than ME/CFS. This region registers task history and uses the information for reorientation to update and shift attention to relevant stimuli such as the sequences of letters viewed in the N-Back tasks (Taylor *et al.*, 2011; Seghier, 2013). The right angular gyrus may have editing functions, because it was strongly involved during the inhibition of inappropriate responses in a variety of go/no-go tasks (Nee *et al.*, 2007).

Exercise had been predicted to improve executive function based on studies in healthy humans, where activation of the dorsolateral prefrontal cortex led to active suppression of attention directed towards previously acquired information, mental representations and tasks. The inhibition of task-irrelevant information (cognitive inhibition) allows new tasks to be performed with greater efficiency (Corbetta and Shulman, 2002; Chang *et al.*, 2012; Ludyga *et al.*, 2016). Dorsolateral frontal regions were not differentially activated in the ME/CFS or GWI groups in this study, suggesting that mechanism(s) of cognitive inhibition were intact in all subjects.

Post-exertional dysfunction of the dorsal attention network (Corbetta and Shulman, 2002) was inferred for GWI because of the decreased activation in the right intraparietal sulcus after exercise relative to HC and ME/CFS subjects. This ROI is required for maintaining attention to salient cues for task completion (Majerus *et al.*, 2012), suggesting that exercise reduced the attention needed to perform the task. The reduction in concentration or focus following exercise may be a cognitive component of post-exertional malaise in GWI. However, caution is required to avoid false positive claims because slight shifts in the BOLD signals for GWI versus ME/CFS may have led to the apparent statistical significance found after exercise.

Anxious individuals have excessive activation of the left, but not the right, posterior parietal cortex, inferior parietal lobule and intraparietal sulcus during a working memory task using Eckman faces (Ekman, 1993) as distractors (Stout *et al.*, 2017). The activation was associated with misallocation of working memory resources, dispositional anxiety and perceived threat. These dysfunctional effects were unlikely to contribute to the cognitive aspects of exertional exhaustion in GWI subjects because they had relative deactivation of the left cuneus/precuneus and right intraparietal sulcus regions after exercise, or in ME/CFS who had right parietal alterations. Left parietal regions were differentially activated in GWI veterans during the Stroop test (Wylie *et al.*, 2019).

Exercise had two predominant effects on brain activity in neural substrates of working memory and cognitive function. In GWI, exercise caused a reduction in BOLD signal in the posterior midbrain, but in ME/CFS it caused an increase in operculo-insular cortical activation.

Exercise either increased activation or deterred deactivation of BOLD signal in the right middle insula and left Rolandic operculum during the cognitive task in the ME/CFS group. This activity may be interpreted as a component of post-exertional malaise with increased perception of bodily discomfort while attempting to maintain attention during the 2-Back cognitive challenge. The right middle insula is a somatosensory homunculus for interoceptive sensation for modalities such as pain, temperature, forced respiration, isometric exercise, itching after cutaneous histamine injection, heart rate awareness and distension of the oesophagus, stomach, bladder and rectum (Craig, 2002; Ronchi *et al.*, 2015; Schulz, 2016; Avery *et al.*, 2017; Evrard, 2019). This region has increased connectivity to the right middle and posterior cingulate cortex in fibromyalgia suggesting involvement in chronic pain (Ichesco *et al.*, 2014). Conversely, left insula activation has been linked to anxiety (Caseras *et al.*, 2013).

The Rolandic operculum corresponds to the junction of the posterior insula, inferior frontal sulcus, and the inferior precentral sulcus. The inferior frontal junction is involved in maintenance of vigilant attention during simple tasks such as the stimulus-response 0-Back task and discrimination tasks that require continuous decisions about alternative responses (e.g. go vs. no-go tasks) (Langner and Eickhoff, 2013). The left inferior frontal junction is dominant for implementing rules for mapping connections between target stimuli and motor responses in discrimination tasks (Hartstra *et al.*, 2010), and for preparing responses to stimuli that are viewed at regular intervals (i.e. fixed as opposed to unpredictable temporal event sequences). Activation may indicate covert or overt verbal rehearsal (Friederici, 2002) as suggested from studies of patients with brain deficits (Alexander *et al.*, 2005). For comparison, the right inferior frontal junction may sustain continuous sensorimotor responses during tasks, and may be vigilant for infrequent but action-relevant signals as part of the right hemisphere ventral attention network (Kucyi *et al.*, 2012). Alternatively, this region may correspond to the posterior insula node in a posterior salience network identified during task (Shirer *et al.*, 2012). The bilateral Rolandic operculum integrates exteroceptive and interoceptive signals that are necessary for bodily self-consciousness and interoceptive awareness as in thermal pain and heartbeat awareness (Wager *et al.*, 2013; Blefari *et al.*, 2017).

Prior to exercise, BOLD signal in the left Rolandic operculum was negative for the 2-Back > 0-Back condition suggesting that all groups had greater activation during the 0-Back task that became relatively reduced during the more demanding 2-Back task. One explanation is that the perceptions of interoceptive awareness became inhibited during the 2-Back task when cognitive resources were needed for concentration and working memory, and distractions had to be inhibited. The same level of relative deactivation was maintained after exercise in the HC and GWI groups. However, in ME/CFS, exertion caused the BOLD signal to remain at baseline, suggesting relative equivalence of activation during the 0-Back and 2-Back tasks. This lack of deactivation may indicate distraction with an elevation of interoceptive and nociceptive awareness during the 2-Back task, selective recruitment of the left Rolandic operculum to maintain vigilant attention as a component of cognitive compensation, or exercise-induced loss of some regulatory function that is required to suppress insular activity during the 2-Back task.

The midbrain ROI has special implications for pain, negative emotion and neurobehavioural dysfunction in ME/CFS and GWI. Recent studies of brain development and prosomeric genoarchitectonics have led to new perspectives into the origins and nomenclature of midbrain and hindbrain structures that have been embraced by the 2017 Terminological Neuroanatomica of the Federative International Programme for Anatomical Terminology (Anatomists; Puelles, 2016, 2019; Watson *et al.*, 2019). The diencephalon forms in response to dorsal–ventral gradients of Pax6 and Otx2. Otx2 directs formation of the midbrain with its widened dorsal but narrow ventral aspect and oculomotor nuclei. The isthmus of the rostral hindbrain forms in response to the dorsal-ventral gradient of Gbx2. The vermis and hemispheres of the cerebellum develop from the dorsal isthmus (isthmocerebellar hindbrain). Embryonic cells in the border of the midbrain and isthmus release Fgf8 that diffuses to form gradients in rostral and caudal directions (Harada *et al.*, 2016). These growth factors may imprint gene expression making the dorsal midbrain susceptible to as yet uncharacterized toxic molecular injuries that lead to distinct deficits or neurobehavioural patterns in GWI and ME/CFS.

The rostral part of the midbrain ROI intersected with Ascending Arousal Network nuclei that were based on histological sections and diffusion studies of white matter tracts (Edlow *et al.*, 2012). The ROI extended from the left to right periaqueductal gray (PAG) and to the adjacent right midbrain reticular formation, inferior colliculus and lateral lemniscus. It continued caudally to the right lateral isthmus. These nuclei have particular relevance to ME/CFS and GWI.

The PAG is a profuse plexus of small and medium sized neurons and largely unmyelinated fibres. It has subdivisions with distinct connectivity for pain modulation, interoception (e.g. dyspnoea) and executive functions (Coulombe *et al.*, 2016; Faull and Pattinson, 2017). Its dorsal columns convey efferent axons from the ‘medial defense zone’ made up of premammillary nuclei, amygdala, stria terminalis, hippocampus and lateral septum (Canteras, 2002) that has been implicated in survival, threat analysis, flight responses, and models of anxiety, panic disorder and rumination (Blanchard *et al.*, 2011; Blanchard, 2017). These are instinctive behavioural and social reactions that drive emotional experience and physiology in response to threats, and provide motivations for hunting, foraging, sexual and maternal actions. The PAG processes and integrates pain, negative emotions and autonomic control (Kober *et al.*, 2008; Wager *et al.*, 2009; Linnman *et al.*, 2012; Buhle *et al.*, 2013; Harricharan *et al.*, 2016), and mediates vigilance, arousal and life and death decisions about imminent predator–prey interactions. Studies in rodents highlight the role of the rostrolateral PAG in hunting behaviours, and functional differences along dorsal–ventral and rostral–caudal axes associated with defensive behaviours (Franklin, 2019). Predator-like behavioural responses lead to social superiority displays or attacks on edible prey. Prey-like behaviours include danger detection (i.e. risk assessment) and contemplation of escape strategies. Detection of a threat activates the PAG and midbrain reticular formation and causes a transition from relaxed wakefulness to high general attention (Kinomura *et al.*, 1996) that allows for focused interrogation of threats (e.g. freezing in place) and the proximity of danger (Mobbs *et al.*, 2007). Dorsolateral and lateral PAG subdivisions activate the sympathetic nervous system, causing hyperarousal symptoms and switching to task preparation and execution. The PAG stimulates descending anti-nociceptive pathways to induce systemic analgesia (Heinricher *et al.*, 2009). Dysfunction of the PAG may contribute to diverse deleterious effects in anxiety, PTSD (Harricharan *et al.*, 2016), chronic pain (La Cesa *et al.*, 2014) and autonomic disorders (Naegeli *et al.*, 2018).

The inferior colliculus is the target of the ascending auditory (lateral lemniscus) and somatosensory pathways from the pons and medulla. This information is then integrated and relayed to the superior colliculus, pretectum and medial geniculate body of the thalamus. Stimulation leads to hyperarousal and aversive behaviours such as the startle response (Brandao *et al.*, 1993; Heeringa and van Dijk, 2016; Xiong *et al.*, 2017) that is accentuated in ME/CFS, GWI and PTSD (Orr *et al.*, 1995).

The midbrain reticular formation is adjacent to spinothalamic, trigeminothalamic and medial lemniscus pathways. It extends caudally into the isthmus as the cuneiform area. In humans, it is activated during the transition from a relaxed awake state to an attention-demanding state during reaction-time tasks (Kinomura *et al.*, 1996). Normal inter-subject variations in T1wSE (myelin and/or iron) in the cuneiform nucleus yielded abnormal correlations with an autonomic measure in ME/CFS suggesting impaired communication with medulla, hypothalamus and other nuclei in the central autonomic network (Barnden *et al.*, 2016). Impaired connectivity between medulla and midbrain reticular formation nuclei was recently confirmed in ME/CFS (Barnden *et al.*, 2019).

This pioneer study was built from numerous stratified statistical contrasts between groups and pre- versus post-exercise scans in order to discover the range of ROIs involved in cognition that were affected by exercise. Multiple comparisons were controlled by statistical modelling in SPM12, *P* < 0.05 FWE and *post hoc* evaluation of significant ANOVAs using Tukey’s HSD without re-testing the same statistic twice (‘double dipping’) (Kriegeskorte *et al.*, 2009; Button, 2019). Values for BOLD signals in each ROI and individual were extracted to calculate effect sizes that could be used to estimate sample sizes for future replication studies. Future studies on smaller groups of subjects (Smith and Little, 2018) may still be of value if their results are compiled with additional published data and analysed by Bayesian hypotheses or using a meta-analytical strategy (Ellis, 2010). Effects sizes were independently analysed by NeuroPowerTools. Sample size estimates were in the 134–157 range for the various ROIs. These sizes translated to Cohen’s d of about 0.4 for equal groups sizes, power of 80% and *P* = 0.05. These effect sizes are useful for the continuous N-Back task, but may not be predictive for Stroop or other cognitive tasks that interrogate other properties of working memory and cognition in ME/CFS (Shan *et al.*, 2018).

The predominance of females in ME/CFS and males in GWI may have contributed to differences in BOLD activation during the working memory task based on a meta-analysis in BrainMap (Hill *et al.*, 2014). This could account for the higher activation of the right angular gyrus before exercise in GWI compared to ME/CFS because males utilize a network with more parietal regions. However, regions that were different between ME/CFS and GWI following exercise were not identified in that meta-analysis, and so the post-exertional effects were unlikely to be attributed to gender. In addition, age and gender were included in the statistical modelling.

Limitations to this study include the need to correct for motion artefacts related to cardiac and respiratory pulsations (Linnman *et al.*, 2012; Buhle *et al.*, 2013; Terem *et al.*, 2018; VanElzakker *et al.*, 2019). Future studies should include heart rate and respiratory rate monitors and processing to reduce the motion artefacts induced by arterial pulsations in the Circle of Willis and vertebrobasal arteries, and changes in venous engorgement related to the positive and negative intrapulmonary pressures during breathing.

This study has several clinical implications. The embryological origins of midbrain, isthmus and cerebellar vermis and hemispheres makes it possible these this brain region have unique gene expression that may allow susceptibility to neurotoxic agents such as those used in the Gulf War. The IIIrd nerve and Edinger–Westphal nucleus are ventral to the midbrain ROI. If dysfunctional, then detailed physical examination of ocular reflexes may provide physical signs for diagnosis of GWI or ME/CFS. This is predicted based on the difficulties with accommodation and blurring that are complaints in both diseases.

The right angular gyrus was the only region to show a difference in activation during the cognitive task prior to the exertional provocation. HC, ME/CFS and GWI responses were equivalent for all other whole brain and specific ROI contrasts suggesting comparable cognitive function at baseline.

Exercise caused significant changes based on the general trend of incrementally increased BOLD activation between the pre- and post-exercise scans in ME/CFS, but decreased activation in GWI. Effect sizes were calculated to plan future investigations of these ROIs using this MRI/continuous N-Back/exercise paradigm. This post-exertional dichotomy suggested that midbrain, insula, and executive systems were dysfunctional in GWI and ME/CFS, but that each disease had distinct responses to exertion. The high prevalences of PTSD and depressive symptoms in GWI may also have contributed to the opposite trends for midbrain activation after exercise in ME/CFS and GWI. Diverse pathophysiological neuromolecular mechanisms may be implicated such as microglial activation (gliosis) and ‘neuroinflammation’, synaptic neurotransmitter plasticity, intracellular signalling systems, regulatory interneuron circuits or modulation of myelination (Barnden *et al.*, 2016, 2018, 2019). If confirmed, then deep brain stimulation of the midbrain, or transcranial direct current stimulation or magnetic stimulation of cerebral cortical regions may be considered as therapeutic options for ME/CFS and GWI in the future.

## Funding

The study was supported by funding from The Sergeant Sullivan Circle, Dr. Barbara Cottone, Dean Clarke Bridge Prize, Department of Defense Congressionally Directed Medical Research Program (CDMRP) W81XWH-15-1-0679 and W81-XWH-09-1-0526, and the National Institute of Neurological Disorders and Stroke RO1NS085131. This project has been funded in whole or in part with Federal funds (Grant #UL1TR000101 previously UL1RR031975) from the National Center for Advancing Translational Sciences (NCATS), National Institutes of Health (NIH), through the Clinical and Translational ScienceAwards Program (CTSA), a trademark of DHHS, part of the Roadmap Initiative, ‘Re-Engineering the Clinical Research Enterprise’.

## Competing Interests

The authors report no competing interests.

## Supplementary Material

fcaa070_Supplementary_Data

## Abbreviations

ΔBOLD =: post-minus pre exercise BOLD response for the 2-Back>0-Back condition
0-back =: stimulus matching task to ‘See a letter, press a button.’
2-Back =: Working memory recall task to view a series of letters and recall each one after a delay of 2 additional letters
2-Back > 0-Back =: difference in BOLD activation between the high cognitive load 2-back task and stimulus matching 0-back task during the N-back working memory task
95% CI =: 95% confidence interval
AAN =: Harvard Ascending Arousal Network atlas
ANOVA =: F-test for differences between any of the 3 groups
BBRAIN =: Boston Biorepository, Recruitment, and Integrative Network for Gulf War Illness
BMI =: body mass index
BOLD =: blood oxygen level dependent signal
CDMRP =: Department of Defense Congressionally Directed Medical Research Program
d =: Cohen’s d measure of effect size for equal sample sizes
EPI =: T2*-weighted gradient-echo planar image
fMRI =: functional magnetic resonance imaging
FOV =: Field of view
Fukuda =: 1984 Center for Disease Control criteria for Chronic Fatigue Syndrome
FWE =: family wise error correction for multiple comparisons
FWHM =: 6 mm full width half maximum
g =: Hedges’ g measure of effect size for unequal sample sizes
GWI =: Gulf War Illness
HC =: healthy controls
HR =: heart rate
HRPO =: Human Research Program Office
HSD =: Tukey’s Honest Significant Difference
IRB =: Institutional Review Board
kE =: Extent threshold for contiguous voxel counts in clusters
ME/CFS =: Myalgic Encephalomyelitis/Chronic Fatigue Syndrome
MNI =: Montreal Neurological Institute
MPRAGE =: structural 3D T1-weighted magnetization-prepared rapid acquisition with gradient echo
PAG =: periaqueductal gray
PPN =: right pendunculotegmental nucleus, formerly pedunculopontine nucleus
PTSD =: post-traumatic stress disorder
ROIs =: regions of interest
SF-36 =: Medical Outcomes Survey Short Form 36 item quality of life questionnaire
SPM12 =: statistical parametric mapping version 12
SUIT =: spatially unbiased atlas template for cerebellum
TE =: echo time
TI =: inversion time
TR =: repetition time

## References

[fcaa070-B1] Alexander MP, Stuss DT, Shallice T, Picton TW, Gillingham S. Impaired concentration due to frontal lobe damage from two distinct lesion sites. Neurology 2005; 65: 572–9. 16116118 10.1212/01.wnl.0000172912.07640.92

[fcaa070-B2] American Psychiatric Association. Diagnostic and statistical manual of mental disorders. 5th edn. Washington, DC: American Psychiatric Publications; 2013.

[fcaa070-B3] Anatomists IFoAo. The Federative International Programme for Anatomical Terminology. Available from: https://fipat.library.dal.ca/tna/ (16 June 2020, date last accessed).

[fcaa070-B4] Armstrong CW, McGregor NR, Butt HL, Gooley PR. Metabolism in chronic fatigue syndrome. Adv Clin Chem 2014; 66: 121–72. 25344988 10.1016/b978-0-12-801401-1.00005-0

[fcaa070-B5] Arslan S, Ktena SI, Makropoulos A, Robinson EC, Rueckert D, Parisot S. Human brain mapping: a systematic comparison of parcellation methods for the human cerebral cortex. Neuroimage 2018; 170: 5–30. 28412442 10.1016/j.neuroimage.2017.04.014

[fcaa070-B6] Avery JA, Gotts SJ, Kerr KL, Burrows K, Ingeholm JE, Bodurka J, et al Convergent gustatory and viscerosensory processing in the human dorsal mid-insula. Hum Brain Mapp 2017; 38: 2150–64. 28070928 10.1002/hbm.23510 PMC5575915

[fcaa070-B7] Baraniuk JN. BMJ Best Practice ME/CFS. 2019 Available from: https://bestpractice.bmj.com/topics/en-us/277 (16 June 2020, date last accessed).

[fcaa070-B8] Baraniuk JN, Adewuyi O, Merck SJ, Ali M, Ravindran MK, Timbol CR, et al A Chronic Fatigue Syndrome (CFS) severity score based on case designation criteria. Am J Transl Res 2013; 5: 53–68. 23390566 PMC3560481

[fcaa070-B9] Baraniuk JN, Shivapurkar N. Exercise-induced changes in cerebrospinal fluid miRNAs in Gulf War Illness, chronic fatigue syndrome and sedentary control subjects. Sci Rep 2017; 7: 15338. 29127316 10.1038/s41598-017-15383-9 PMC5681566

[fcaa070-B10] Barnden LR, Kwiatek R, Crouch B, Burnet R, Del Fante P. Autonomic correlations with MRI are abnormal in the brainstem vasomotor centre in chronic fatigue syndrome. Neuroimage Clin 2016; 11: 530–7. 27114901 10.1016/j.nicl.2016.03.017 PMC4833047

[fcaa070-B11] Barnden LR, Shan ZY, Staines DR, Marshall-Gradisnik S, Finegan K, Ireland T, et al Hyperintense sensorimotor T1 spin echo MRI is associated with brainstem abnormality in chronic fatigue syndrome. Neuroimage Clin 2018; 20: 102–9. 30497131 10.1016/j.nicl.2018.07.011 PMC6309570

[fcaa070-B12] Barnden LR, Shan ZY, Staines DR, Marshall-Gradisnik S, Finegan K, Ireland T, et al Intra brainstem connectivity is impaired in chronic fatigue syndrome. Neuroimage Clin 2019; 24: 102045. 31671321 10.1016/j.nicl.2019.102045 PMC6835065

[fcaa070-B13] Blanchard DC. Translating dynamic defense patterns from rodents to people. Neurosci Biobehav Rev 2017; 76: 22–8. 28434585 10.1016/j.neubiorev.2016.11.001

[fcaa070-B14] Blanchard DC, Griebel G, Pobbe R, Blanchard RJ. Risk assessment as an evolved threat detection and analysis process. Neurosci Biobehav Rev 2011; 35: 991–8. 21056591 10.1016/j.neubiorev.2010.10.016

[fcaa070-B15] Blefari ML, Martuzzi R, Salomon R, Bello-Ruiz J, Herbelin B, Serino A, et al Bilateral Rolandic operculum processing underlying heartbeat awareness reflects changes in bodily self-consciousness. Eur J Neurosci 2017; 45: 1300–12. 28370498 10.1111/ejn.13567

[fcaa070-B16] Bohland JW, Bokil H, Allen CB, Mitra PP. The brain atlas concordance problem: quantitative comparison of anatomical parcellations. PLoS One 2009; 4: e7200. 19787067 10.1371/journal.pone.0007200 PMC2748707

[fcaa070-B17] Brandao ML, Melo LL, Cardoso SH. Mechanisms of defense in the inferior colliculus. Behav Brain Res 1993; 58: 49–55. 8136049 10.1016/0166-4328(93)90089-9

[fcaa070-B18] Brett M, Anton J-L, Valabregue R, Poline J, Region of interest analysis using an SPM toolbox. In: 8th International Conference on Functional Mapping of the Human Brain; 2002; Sendai, Japan: Sendai, Japan; 2002.

[fcaa070-B19] Buhle JT, Kober H, Ochsner KN, Mende-Siedlecki P, Weber J, Hughes BL, et al Common representation of pain and negative emotion in the midbrain periaqueductal gray. Soc Cogn Affect Neurosci 2013; 8: 609–16. 22446299 10.1093/scan/nss038 PMC3739905

[fcaa070-B20] Button KS. Double-dipping revisited. Nat Neurosci 2019; 22: 688–90. 31011228 10.1038/s41593-019-0398-z

[fcaa070-B21] Caligiuri M, Murray C, Buchwald D, Levine H, Cheney P, Peterson D, et al Phenotypic and functional deficiency of natural killer cells in patients with chronic fatigue syndrome. J Immunol 1987; 139: 3306–13. 2824604

[fcaa070-B22] Canteras NS. The medial hypothalamic defensive system: hodological organization and functional implications. Pharmacol Biochem Behav 2002; 71: 481–91. 11830182 10.1016/s0091-3057(01)00685-2

[fcaa070-B23] Carruthers BM, Jain AK, De Meirleir KL, Peterson DL, Klimas NG, Lerner AM, et al Myalgic encephalomyelitis/chronic fatigue syndrome. J Chronic Fatig Syndr 2003; 11: 7–115.

[fcaa070-B24] Caseras X, Murphy K, Mataix-Cols D, Lopez-Sola M, Soriano-Mas C, Ortriz H, et al Anatomical and functional overlap within the insula and anterior cingulate cortex during interoception and phobic symptom provocation. Hum Brain Mapp 2013; 34: 1220–9. 22162203 10.1002/hbm.21503 PMC6869871

[fcaa070-B25] Cella M, Chalder T. Measuring fatigue in clinical and community settings. J Psychosom Res 2010; 69: 17–22. 20630259 10.1016/j.jpsychores.2009.10.007

[fcaa070-B26] Chang YK, Labban JD, Gapin JI, Etnier JL. The effects of acute exercise on cognitive performance: a meta-analysis. Brain Res 2012; 1453: 87–101. 22480735 10.1016/j.brainres.2012.02.068

[fcaa070-B27] Cohen J, Statistical power analysis for the behavioral sciences. 2nd ed. Hillsdale, NJ: Erlbaum; 1988.

[fcaa070-B28] Corbetta M, Shulman GL. Control of goal-directed and stimulus-driven attention in the brain. Nat Rev Neurosci 2002; 3: 201–15. 11994752 10.1038/nrn755

[fcaa070-B29] Coulombe MA, Erpelding N, Kucyi A, Davis KD. Intrinsic functional connectivity of periaqueductal gray subregions in humans. Hum Brain Mapp 2016; 37: 1514–30. 26821847 10.1002/hbm.23117 PMC6867375

[fcaa070-B30] Craig AD. How do you feel? Interoception: the sense of the physiological condition of the body. Nat Rev Neurosci 2002; 3: 655–66. 12154366 10.1038/nrn894

[fcaa070-B31] Daugherty SA, Henry BE, Peterson DL, Swarts RL, Bastien S, Thomas RS. Chronic fatigue syndrome in northern Nevada. Rev Infect Dis 1991; 13 Suppl 1: S39–44. 1850542 10.1093/clinids/13.supplement_1.s39

[fcaa070-B32] Diedrichsen J. A spatially unbiased atlas template of the human cerebellum. Neuroimage 2006; 33: 127–38. 16904911 10.1016/j.neuroimage.2006.05.056

[fcaa070-B33] Diedrichsen J, Balsters JH, Flavell J, Cussans E, Ramnani N. A probabilistic MR atlas of the human cerebellum. Neuroimage 2009; 46: 39–46. 19457380 10.1016/j.neuroimage.2009.01.045

[fcaa070-B34] Du Preez S, Corbitt M, Cabanas H, Eaton N, Staines D, Marshall-Gradisnik S. A systematic review of enteric dysbiosis in chronic fatigue syndrome/myalgic encephalomyelitis. Syst Rev 2018; 7: 241. 30572962 10.1186/s13643-018-0909-0 PMC6302292

[fcaa070-B35] Durnez J, Degryse J, Moerkerke B, Seurinck R, Sochat V, Poldrack RA, et al Power and sample size calculations for fMRI studies based on the prevalence of active peaks. bioRxiv 2016.

[fcaa070-B36] Dworkin RH, Turk DC, Revicki DA, Harding G, Coyne KS, Peirce-Sandner S, et al Development and initial validation of an expanded and revised version of the Short-form McGill Pain Questionnaire (SF-MPQ-2). Pain 2009; 144: 35–42. 19356853 10.1016/j.pain.2009.02.007

[fcaa070-B37] Edlow BL, Takahashi E, Wu O, Benner T, Dai G, Bu L, et al Neuroanatomic connectivity of the human ascending arousal system critical to consciousness and its disorders. J Neuropathol Exp Neurol 2012; 71: 531–46. 22592840 10.1097/NEN.0b013e3182588293 PMC3387430

[fcaa070-B38] Eisen SA, Kang HK, Murphy FM, Blanchard MS, Reda DJ, Henderson WG, et al Gulf War veterans’ health: medical evaluation of a U.S. cohort. Ann Intern Med 2005; 142: 881–90. 15941694 10.7326/0003-4819-142-11-200506070-00005

[fcaa070-B39] Ekman P. Facial expression and emotion. Am Psychol 1993; 48: 384–92. 8512154 10.1037//0003-066x.48.4.384

[fcaa070-B40] Ellis PD. The essential guide to effect sizes: Statistical power, Meta-Analysis, and the interpretation of research results. Cambridge, UK: Cambridge University Press; 2010.

[fcaa070-B41] Evans AC, Collins DL, Mills SR, Brown ED, Kelly RL, Peters TM, 3D statistical neuroanatomical models from 305 MRI volumes. In: Proceedings of IEEE-Nuclear Science Symposium and Medical Imaging Conference 1993, p. 1813–7.

[fcaa070-B42] Evrard HC. The organization of the primate insular cortex. Front Neuroanat 2019; 13: 43. 31133822 10.3389/fnana.2019.00043 PMC6517547

[fcaa070-B43] Faull OK, Pattinson KT. The cortical connectivity of the periaqueductal gray and the conditioned response to the threat of breathlessness. Elife 2017; 6. 10.7554/eLife.21749 PMC5332157 28211789

[fcaa070-B44] Franklin TB. Recent advancements surrounding the role of the periaqueductal gray in predators and prey. Front Behav Neurosci 2019; 13: 60. 31133827 10.3389/fnbeh.2019.00060 PMC6524621

[fcaa070-B45] Friedberg F, Bateman L, Bested A, Davenport T, Friedman K, Gurwitt A, et al Chronic fatigue syndrome/myalgic encephalomyelitis: a primer for clinical practitioners. Scottsdale, AZ: Wilshire Press; 2014.

[fcaa070-B46] Friederici AD. Towards a neural basis of auditory sentence processing. Trends Cogn Sci 2002; 6: 78–84. 15866191 10.1016/s1364-6613(00)01839-8

[fcaa070-B47] Friston KJ, Williams S, Howard R, Frackowiak RS, Turner R. Movement-related effects in fMRI time-series. Magn Reson Med 1996; 35: 346–55. 8699946 10.1002/mrm.1910350312

[fcaa070-B48] Fukuda K, Nisenbaum R, Stewart G, Thompson WW, Robin L, Washko RM, et al Chronic multisymptom illness affecting Air Force veterans of the Gulf War. JAMA 1998; 280: 981–8. 9749480 10.1001/jama.280.11.981

[fcaa070-B49] Fukuda K, Straus SE, Hickie I, Sharpe MC, Dobbins JG, Komaroff A. The chronic fatigue syndrome: a comprehensive approach to its definition and study. International Chronic Fatigue Syndrome Study Group. Ann Intern Med 1994; 121: 953–9. 7978722 10.7326/0003-4819-121-12-199412150-00009

[fcaa070-B50] Garner RS, Rayhan RU, Baraniuk JN. Verification of exercise-induced transient postural tachycardia phenotype in Gulf War Illness. Am J Transl Res 2018; 10: 3254–64. 30416666 PMC6220213

[fcaa070-B51] Harada H, Sato T, Nakamura H. Fgf8 signaling for development of the midbrain and hindbrain. Develop Growth Differ 2016; 58: 437–45. 10.1111/dgd.12293 27273073

[fcaa070-B52] Harricharan S, Rabellino D, Frewen PA, Densmore M, Theberge J, McKinnon MC, et al fMRI functional connectivity of the periaqueductal gray in PTSD and its dissociative subtype. Brain Behav 2016; 6: e00579. 28032002 10.1002/brb3.579 PMC5167004

[fcaa070-B53] Hartstra E, Oldenburg JF, Van Leijenhorst L, Rombouts SA, Crone EA. Brain regions involved in the learning and application of reward rules in a two-deck gambling task. Neuropsychologia 2010; 48: 1438–46. 20105435 10.1016/j.neuropsychologia.2010.01.012

[fcaa070-B54] Hedges LV. Distribution theory for Glass’s estimator of effect size and related estimators. J Educ Stat 1981; 6: 107–28.

[fcaa070-B55] Heeringa AN, van Dijk P. The immediate effects of acoustic trauma on excitation and inhibition in the inferior colliculus: a Wiener-kernel analysis. Hear Res 2016; 331: 47–56. 26523371 10.1016/j.heares.2015.10.007

[fcaa070-B56] Heinricher MM, Tavares I, Leith JL, Lumb BM. Descending control of nociception: specificity, recruitment and plasticity. Brain Res Rev 2009; 60: 214–25. 19146877 10.1016/j.brainresrev.2008.12.009 PMC2894733

[fcaa070-B57] Hill AC, Laird AR, Robinson JL. Gender differences in working memory networks: a BrainMap meta-analysis. Biol Psychol 2014; 102: 18–29. 25042764 10.1016/j.biopsycho.2014.06.008 PMC4157091

[fcaa070-B58] Horn A, Kuhn AA. Lead-DBS: a toolbox for deep brain stimulation electrode localizations and visualizations. Neuroimage 2015; 107: 127–35. 25498389 10.1016/j.neuroimage.2014.12.002

[fcaa070-B59] Horn A, Li N, Dembek TA, Kappel A, Boulay C, Ewert S, et al Lead-DBS v2: towards a comprehensive pipeline for deep brain stimulation imaging. Neuroimage 2019; 184: 293–316. 30179717 10.1016/j.neuroimage.2018.08.068 PMC6286150

[fcaa070-B60] Hornig M, Gottschalk G, Peterson DL, Knox KK, Schultz AF, Eddy ML, et al Cytokine network analysis of cerebrospinal fluid in myalgic encephalomyelitis/chronic fatigue syndrome. Mol Psychiatry 2016; 21: 261–9. 25824300 10.1038/mp.2015.29

[fcaa070-B61] Ichesco E, Schmidt-Wilcke T, Bhavsar R, Clauw DJ, Peltier SJ, Kim J, et al Altered resting state connectivity of the insular cortex in individuals with fibromyalgia. J Pain 2014; 15: 815–26 e1. 24815079 10.1016/j.jpain.2014.04.007 PMC4127388

[fcaa070-B62] Jones JF, Lin JM, Maloney EM, Boneva RS, Nater UM, Unger ER, et al An evaluation of exclusionary medical/psychiatric conditions in the definition of chronic fatigue syndrome. BMC Med 2009; 7: 57. 19818157 10.1186/1741-7015-7-57 PMC2768736

[fcaa070-B63] Keech A, Sandler CX, Vollmer-Conna U, Cvejic E, Lloyd AR, Barry BK. Capturing the post-exertional exacerbation of fatigue following physical and cognitive challenge in patients with chronic fatigue syndrome. J Psychosom Res 2015; 79: 537–49. 26359713 10.1016/j.jpsychores.2015.08.008

[fcaa070-B64] Kinomura S, Larsson J, Guly S B. Z, Roland PE. Activation by attention of the human reticular formation and thalamic intralaminar nuclei. Science 1996; 271: 512–5. 8560267 10.1126/science.271.5248.512

[fcaa070-B65] Kober H, Barrett LF, Joseph J, Bliss-Moreau E, Lindquist K, Wager TD. Functional grouping and cortical-subcortical interactions in emotion: a meta-analysis of neuroimaging studies. Neuroimage 2008; 42: 998–1031. 18579414 10.1016/j.neuroimage.2008.03.059 PMC2752702

[fcaa070-B67] Kriegeskorte N, Simmons WK, Bellgowan PS, Baker CI. Circular analysis in systems neuroscience: the dangers of double dipping. Nat Neurosci 2009; 12: 535–40. 19396166 10.1038/nn.2303 PMC2841687

[fcaa070-B68] Kucyi A, Hodaie M, Davis KD. Lateralization in intrinsic functional connectivity of the temporoparietal junction with salience- and attention-related brain networks. J Neurophysiol 2012; 108: 3382–92. 23019004 10.1152/jn.00674.2012

[fcaa070-B69] La Cesa S, Tinelli E, Toschi N, Di Stefano G, Collorone S, Aceti A, et al fMRI pain activation in the periaqueductal gray in healthy volunteers during the cold pressor test. Magn Reson Imaging 2014; 32: 236–40. 24468081 10.1016/j.mri.2013.12.003

[fcaa070-B70] Langner R, Eickhoff SB. Sustaining attention to simple tasks: a meta-analytic review of the neural mechanisms of vigilant attention. Psychol Bull 2013; 139: 870–900. 23163491 10.1037/a0030694 PMC3627747

[fcaa070-B71] Linnman C, Moulton EA, Barmettler G, Becerra L, Borsook D. Neuroimaging of the periaqueductal gray: state of the field. Neuroimage 2012; 60: 505–22. 22197740 10.1016/j.neuroimage.2011.11.095 PMC3288184

[fcaa070-B72] Ludyga S, Gerber M, Brand S, Holsboer-Trachsler E, Puhse U. Acute effects of moderate aerobic exercise on specific aspects of executive function in different age and fitness groups: a meta-analysis. Psychophysiology 2016; 53: 1611–26. 27556572 10.1111/psyp.12736

[fcaa070-B73] Majerus S, Attout L, D'Argembeau A, Degueldre C, Fias W, Maquet P, et al Attention supports verbal short-term memory via competition between dorsal and ventral attention networks. Cereb Cortex 2012; 22: 1086–97. 21765184 10.1093/cercor/bhr174

[fcaa070-B74] Mawson AR, Croft AM. Gulf War Illness: unifying hypothesis for a continuing health problem. Int J Environ Res Public Health 2019; 16. 10.3390/ijerph16010111 PMC6339135 30609834

[fcaa070-B75] Mehalick ML, Schmaling KB, Sabath DE, Buchwald DS. Longitudinal associations of lymphocyte subsets with clinical outcomes in chronic fatigue syndrome. Fatigue 2018; 6: 80–91. 30112249 10.1080/21641846.2018.1426371 PMC6089525

[fcaa070-B76] Mobbs D, Petrovic P, Marchant JL, Hassabis D, Weiskopf N, Seymour B, et al When fear is near: threat imminence elicits prefrontal-periaqueductal gray shifts in humans. Science 2007; 317: 1079–83. 17717184 10.1126/science.1144298 PMC2648508

[fcaa070-B77] Naegeli C, Zeffiro T, Piccirelli M, Jaillard A, Weilenmann A, Hassanpour K, et al Locus coeruleus activity mediates hyperresponsiveness in posttraumatic stress disorder. Biol Psychiatry 2018; 83: 254–62. 29100627 10.1016/j.biopsych.2017.08.021

[fcaa070-B78] Nakatomi Y, Mizuno K, Ishii A, Wada Y, Tanaka M, Tazawa S, et al Neuroinflammation in patients with chronic fatigue syndrome/myalgic encephalomyelitis: an (1)(1)C-(R)-PK11195 PET study. J Nucl Med 2014; 55: 945–50. 24665088 10.2967/jnumed.113.131045

[fcaa070-B79] Naranch K, Park YJ, Repka-Ramirez MS, Velarde A, Clauw D, Baraniuk JN. A tender sinus does not always mean rhinosinusitis. Otolaryngol Head Neck Surg 2002; 127: 387–97. 12447232 10.1067/mhn.2002.129038

[fcaa070-B80] Nee DE, Wager TD, Jonides J. Interference resolution: insights from a meta-analysis of neuroimaging tasks. Cogn Affect Behav Neurosci 2007; 7: 1–17. 17598730 10.3758/cabn.7.1.1

[fcaa070-B81] Orr SP, Lasko NB, Shalev AY, Pitman RK. Physiologic responses to loud tones in Vietnam veterans with posttraumatic stress disorder. J Abnorm Psychol 1995; 104: 75–82. 7897056 10.1037//0021-843x.104.1.75

[fcaa070-B82] Owen AM, McMillan KM, Laird AR, Bullmore E. N-back working memory paradigm: a meta-analysis of normative functional neuroimaging studies. Hum Brain Mapp 2005; 25: 46–59. 15846822 10.1002/hbm.20131 PMC6871745

[fcaa070-B83] Puelles L. Comments on the limits and internal structure of the mammalian midbrain. Anatomy 2016; 10: 60–70.

[fcaa070-B84] Puelles L. Survey of midbrain, diencephalon, and hypothalamus neuroanatomic terms whose prosomeric definition conflicts with columnar tradition. Front Neuroanat 2019; 13: 20. 30873012 10.3389/fnana.2019.00020 PMC6402269

[fcaa070-B85] Rayhan RU, Stevens BW, Raksit MP, Ripple JA, Timbol CR, Adewuyi O, et al Exercise challenge in Gulf War Illness reveals two subgroups with altered brain structure and function. PLoS One 2013; 8: e63903. 23798990 10.1371/journal.pone.0063903 PMC3683000

[fcaa070-B86] Rayhan RU, Washington SD, Garner R, Zajur K, Martinez Addiego F, VanMeter JW, et al Exercise challenge alters default mode network dynamics in Gulf War Illness. BMC Neurosci 2019; 20: 7. 30791869 10.1186/s12868-019-0488-6 PMC6385399

[fcaa070-B87] Reeves WC, Lloyd A, Vernon SD, Klimas N, Jason LA, Bleijenberg G, et al; International Chronic Fatigue Syndrome Study Group. Identification of ambiguities in the 1994 chronic fatigue syndrome research case definition and recommendations for resolution. BMC Health Serv Res 2003; 3: 25. 14702202 10.1186/1472-6963-3-25 PMC317472

[fcaa070-B88] Ronchi R, Bello-Ruiz J, Lukowska M, Herbelin B, Cabrilo I, Schaller K, et al Right insular damage decreases heartbeat awareness and alters cardio-visual effects on bodily self-consciousness. Neuropsychologia 2015; 70: 11–20. 25676677 10.1016/j.neuropsychologia.2015.02.010

[fcaa070-B89] Rottschy C, Langner R, Dogan I, Reetz K, Laird AR, Schulz JB, et al Modelling neural correlates of working memory: a coordinate-based meta-analysis. Neuroimage 2012; 60: 830–46. 22178808 10.1016/j.neuroimage.2011.11.050 PMC3288533

[fcaa070-B90] Schulz SM. Neural correlates of heart-focused interoception: a functional magnetic resonance imaging meta-analysis. Philos Trans R Soc Lond B Biol Sci 2016; 371: 1708. 10.1098/rstb.2016.0018 PMC5062106 28080975

[fcaa070-B91] Seghier ML. The angular gyrus: multiple functions and multiple subdivisions. Neuroscientist 2013; 19: 43–61. 22547530 10.1177/1073858412440596 PMC4107834

[fcaa070-B92] Shan ZY, Finegan K, Bhuta S, Ireland T, Staines DR, Marshall-Gradisnik SM, et al Brain function characteristics of chronic fatigue syndrome: a task fMRI study. Neuroimage Clin 2018; 19: 279–86. 30035022 10.1016/j.nicl.2018.04.025 PMC6051500

[fcaa070-B93] Shirer WR, Ryali S, Rykhlevskaia E, Menon V, Greicius MD. Decoding subject-driven cognitive states with whole-brain connectivity patterns. Cereb Cortex 2012; 22: 158–65. 21616982 10.1093/cercor/bhr099 PMC3236795

[fcaa070-B94] Sitek KR, Gulban OF, Calabrese E, Johnson GA, Lage-Castellanos A, Moerel M, et al Mapping the human subcortical auditory system using histology, post mortem MRI and in vivo MRI at 7T. Elife 2019; 8: e48932. 10.7554/eLife.48932 PMC6707786 31368891

[fcaa070-B95] Smith PL, Little DR. Small is beautiful: In defense of the small-N design. Psychon Bull Rev 2018; 25: 2083–101. 29557067 10.3758/s13423-018-1451-8 PMC6267527

[fcaa070-B96] Spitzer RL, Kroenke K, Williams JB. Validation and utility of a self-report version of PRIME-MD: the PHQ primary care study. Primary care evaluation of mental disorders. Patient health questionnaire. JAMA 1999; 282: 1737–44. 10568646 10.1001/jama.282.18.1737

[fcaa070-B97] Stangroom J. Social Science Statistics. 2019 Effect Size Calculator for T-Test. Available from: https://www.socscistatistics.com/effectsize/default3.aspx (18 April 2019, date last accessed).

[fcaa070-B98] Steele L. Prevalence and patterns of Gulf War Illness in Kansas veterans: association of symptoms with characteristics of person, place, and time of military service. Am J Epidemiol 2000; 152: 992–1002. 11092441 10.1093/aje/152.10.992

[fcaa070-B99] Stout DM, Shackman AJ, Pedersen WS, Miskovich TA, Larson CL. Neural circuitry governing anxious individuals’ mis-allocation of working memory to threat. Sci Rep 2017; 7: 8742. 28821746 10.1038/s41598-017-08443-7 PMC5562789

[fcaa070-B100] Taylor PC, Muggleton NG, Kalla R, Walsh V, Eimer M. TMS of the right angular gyrus modulates priming of pop-out in visual search: combined TMS-ERP evidence. J Neurophysiol 2011; 106: 3001–9. 21880940 10.1152/jn.00121.2011

[fcaa070-B101] Terem I, Ni WW, Goubran M, Rahimi MS, Zaharchuk G, Yeom KW, et al Revealing sub-voxel motions of brain tissue using phase-based amplified MRI (aMRI). Magn Reson Med 2018; 80: 2549–59. 29845645 10.1002/mrm.27236 PMC6269230

[fcaa070-B102] Tzourio-Mazoyer N, Landeau B, Papathanassiou D, Crivello F, Etard O, Delcroix N, et al Automated anatomical labeling of activations in SPM using a macroscopic anatomical parcellation of the MNI MRI single-subject brain. Neuroimage 2002; 15: 273–89. 11771995 10.1006/nimg.2001.0978

[fcaa070-B103] VanElzakker MB, Brumfield SA, Lara Mejia PS. Corrigendum: Neuroinflammation and cytokines in myalgic encephalomyelitis/chronic fatigue syndrome (ME/CFS): a critical review of research methods. Front Neurol 2019; 10: 316. 31001197 10.3389/fneur.2019.00316 PMC6454267

[fcaa070-B104] Vellage AK, Becke A, Strumpf H, Baier B, Schonfeld MA, Hopf JM, et al Filtering and storage working memory networks in younger and older age. Brain Behav 2016; 6: e00544. 27843697 10.1002/brb3.544 PMC5102642

[fcaa070-B105] Vul E, Harris C, Winkielman P, Pashler H. Puzzlingly high correlations in fMRI studies of emotion, personality, and social cognition. Perspect Psychol Sci 2009; 4: 274–90. 26158964 10.1111/j.1745-6924.2009.01125.x

[fcaa070-B106] Wager TD, Atlas LY, Lindquist MA, Roy M, Woo CW, Kross E. An fMRI-based neurologic signature of physical pain. N Engl J Med 2013; 368: 1388–97. 23574118 10.1056/NEJMoa1204471 PMC3691100

[fcaa070-B107] Wager TD, van Ast VA, Hughes BL, Davidson ML, Lindquist MA, Ochsner KN. Brain mediators of cardiovascular responses to social threat, part II: prefrontal-subcortical pathways and relationship with anxiety. Neuroimage 2009; 47: 836–51. 19465135 10.1016/j.neuroimage.2009.05.044 PMC4169880

[fcaa070-B108] Ware JE, Sherbourne CD. The MOS 36-item short-form health survey (SF-36): I. Conceptual framework and item selection. Med Care 1992; 30: 473–83. 1593914

[fcaa070-B109] Washington SD, Rayhan RU, Garner R, Provenzano D, Zajur K, Martinez Addiego F, et al Exercise alters cerebellar and cortical activity related to working memory in phenotypes of Gulf War Illness. Brain Commun 2020; 2: fcz039. 32025659 10.1093/braincomms/fcz039 PMC6989731

[fcaa070-B110] Watson C, Bartholomaeus C, Puelles L. Time for radical changes in brain stem nomenclature-applying the lessons from developmental gene patterns. Front Neuroanat 2019; 13: 10. 30809133 10.3389/fnana.2019.00010 PMC6380082

[fcaa070-B111] White RF, Steele L, O'Callaghan JP, Sullivan K, Binns JH, Golomb BA, et al Recent research on Gulf War Illness and other health problems in veterans of the 1991 Gulf War: effects of toxicant exposures during deployment. Cortex 2016; 74: 449–75. 26493934 10.1016/j.cortex.2015.08.022 PMC4724528

[fcaa070-B112] Whitefield-Gabrieli S, Nieto-Castanon A. Conn: a functional connectivity toolbox for correlated and anticorrelated brain networks. Brain Connect 2012; 2: 125–41. doi: 10.1089/brain.2012.0073. 22642651

[fcaa070-B113] Wylie GR, Genova H, Dobryakova E, DeLuca J, Chiaravalloti N, Falvo M, et al Fatigue in Gulf War Illness is associated with tonically high activation in the executive control network. Neuroimage Clin 2019; 21: 101641. 30558870 10.1016/j.nicl.2018.101641 PMC6411905

[fcaa070-B114] Xiong B, Alkharabsheh A, Manohar S, Chen GD, Yu N, Zhao X, et al Hyperexcitability of inferior colliculus and acoustic startle reflex with age-related hearing loss. Hear Res 2017; 350: 32–42. 28431308 10.1016/j.heares.2017.03.011 PMC9083438

