# Supplementary material for: Exercise alters brain activation in Gulf War Illness and Myalgic Encephalomyelitis/Chronic Fatigue Syndrome
Source: Brain Commun. 2020 Aug 10;2(2):fcaa070. doi: 10.1093/braincomms/fcaa070 (PMC7425336; doi:10.1093/braincomms/fcaa070)
Supplement: fcaa070_Supplementary_Data [file fcaa070_supplementary_data.pdf]

SUPPLEMENTARY MATERIAL

Exercise alters brain activation in Gulf War Illness and Myalgic Encephalomyelitis / Chronic Fatigue Syndrome

Stuart D. Washington, Rakib U. Rayhan, Richard Garner, Destie Provenzano, Kristina Zajur, Florencia Martinez Addiego, John W. VanMeter, and James N. Baraniuk

Georgetown University, Washington DC

The majority of figures in the main text are derived from two-sample t-tests performed after a One-way ANOVA. Voxel-wise ANOVAs can provide no information about the specific group difference that resulted in clusters nor the relative directions and magnitudes of these differences. Thus, in the main text, our figures are based on results of the two-sample t-tests. From a strictly statistical viewpoint on multiple comparisons, however, it is preferable to present analyses of ROIs resulting from an ANOVA as opposed to those resulting from two-sample t-tests. Here, we report results of analyses of ROIs generated by a voxel-wise One-way ANOVA on the 2-Back>0-Back contrasts across the HC, GWI, and ME/CFS groups after exercise. A similar ANOVA performed on 2-Back>0-Back contrasts across these same groups before exercise yielded no significant clusters.

Left sensorimotor cortex (cluster level: p=0.004, FWE; k_E=180) was one of two ROIs that did not have a voxel overlap with ROIs yielded by subsequent two-sample t-tests. The composition of this ROI per the AAL atlas was left paracentral lobule (32/180, 18%), postcentral gyrus (48/180, 27%), and precentral gyrus (83/180, 46%). The GWI group had significantly less activation in the left sensorimotor cortex than the HC or ME/CFS groups after exercise (Supplementary Figure 1). Specifically, activation levels were significantly higher for HC (p=0.00041, HSD) and ME/CFS (p=0.0041, HSD) relative to GWI. This difference reflects both a decrease in GWI (paired t-test: p=0.039) and an increase in ME/CFS (paired t-test: p=0.036) activation after exercise. Before exercise, there were no differences between groups.

Similarly, the GWI group had significantly less activation in the left cuneus/precuneus ROI (cluster level: p=0.046, FWE; k_E=109) than the HC (p=0.0021, HSD) or ME/CFS (p=0.011, HSD) groups after exercise (Supplementary Figure 2). The composition of this ROI was left cuneus (24/109, 22%), superior occipital gyrus (42/109, 39%), superior parietal gyrus (10/109, 9%), and precuneus (33/109, 30%). As there were no paired differences between GWI and ME/CFS, these differences likely emerged due to relatively small post-exertional increases and decreases in ME/CFS and GWI BOLD activity, respectively. Like the left sensorimotor cortex ROI above, the left cuneus/precuneus had no voxel overlap with ROIs yielded by two-sample t-tests.

Activation in the midbrain/isthmus (cluster level: p=0.040, FWE; k_E=113) ROI greatly decreased after exercise in the GWI group (Supplementary Figure 3). This difference reflects a post-exertional decrease in GWI (paired t-test: p=0.0012) activation alone, as exercise had little effect on HC and ME/CFS activation. Specifically, activation levels were significantly higher for HC (p=0.0024, HSD) and ME/CFS (p=0.00011, HSD) relative to GWI. There were no differences between HC, GWI, and ME/CFS before exercise. As stated in the main text, this ROI partially overlapped with the midbrain ROI yielded from the ME/CFS>GWI contrast.

Likewise, the right intraparietal sulcus (cluster level: p=0.051, FWE; k_E=106) ROI shows differences in activation between the HC, GWI, and ME/CFS groups after exercise (Supplementary Figure 4). As this ROI results from a statistical trend, it is inappropriate to apply Tukey's HSD values to these ROI analyses. However, paired comparisons of BOLD activity elicited before and after exercise are still informative, showing that activation dropped after exercise in the GWI group (paired t-test: p=0.029) but increased in ME/CFS (paired t-test: p=0.013). As stated in the main text, this ROI completely overlapped with a right intraparietal sulcus ROI resulting from the ME/CFS>GWI contrast.

Lastly, the left Rolandic operculum (cluster level: p=0.066, FWE; k_E=99) ROI shows differences in activation between the HC, GWI, and ME/CFS groups after exercise, despite having quite a different response profile from the ROIs above (Supplementary Figure 5). There were no differences between HC, GWI, and ME/CFS before exercise, as all three groups displayed a relatively high magnitude deactivation within this region. After exercise, both HC and GWI continued to display relative deactivation whereas the ME/CFS group failed to deactivate. Again, as this ROI results from a statistical trend, Tukey's HSD values are inappropriate to report. However, paired comparisons of BOLD activity before and after exercise reveal as significant increase in activity within the ME/CFS group alone (paired t-test: p=0.0040). Further, the pattern of activation within this region closely matches that of the 273-voxel Rolandic operculum ROI elicited by the ME/CFS>GWI contrast and is nearly identical to that of the 373-voxel Rolandic operculum ROI elicited by the ME/CFS>HC contrast. There is a high degree of overlap between these three Rolandic operculum ROIs, making similarities between their activation patterns unsurprising.

Supplementary Figure 1.

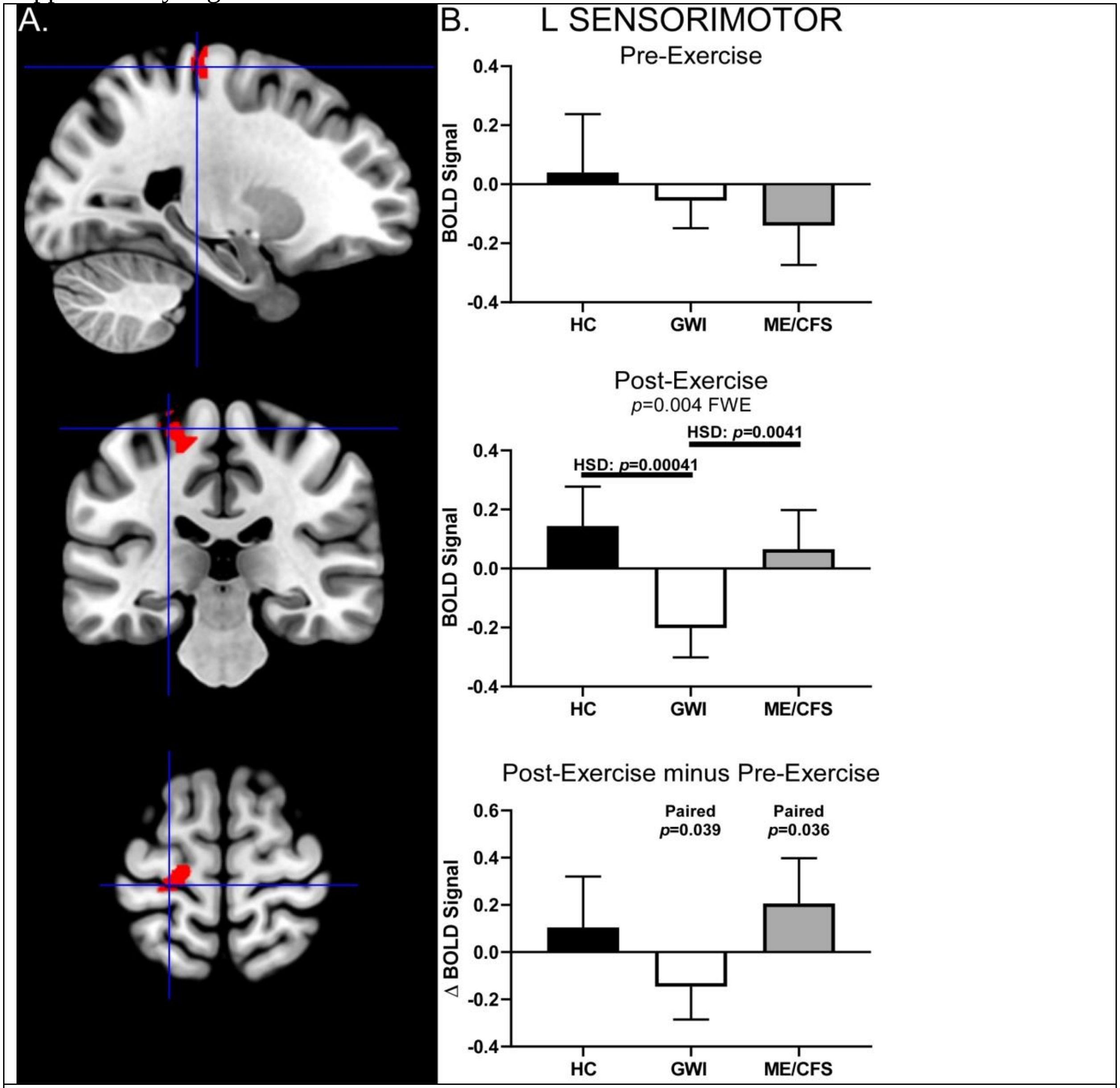

Supplementary Figure 1. Left sensorimotor ROI identified by contrasting BOLD activity derived from the 2-Back>0-Back conditions in the HC, GWI, and ME/CFS groups via One-way ANOVA after exercise (cluster level: p=0.004, FWE; k_E=180). (A) Sagittal (top), coronal (middle), and transverse (bottom) slices of an MNI-standard brain, where crosshairs indicate the cluster's most active voxel (-24, -28, 66). (B) BOLD response for the 2-Back>0-Back condition (mean ± SEM) are shown for pre-exercise (top) and post-exercise (middle). DBOLD (bottom) is the post- minus pre-exercise BOLD response for the 2-Back>0-Back condition for the control (black bars), GWI (white bars), and ME/CFS (gray bars). Error bars represent 95% C.I.

Supplementary Figure 2.

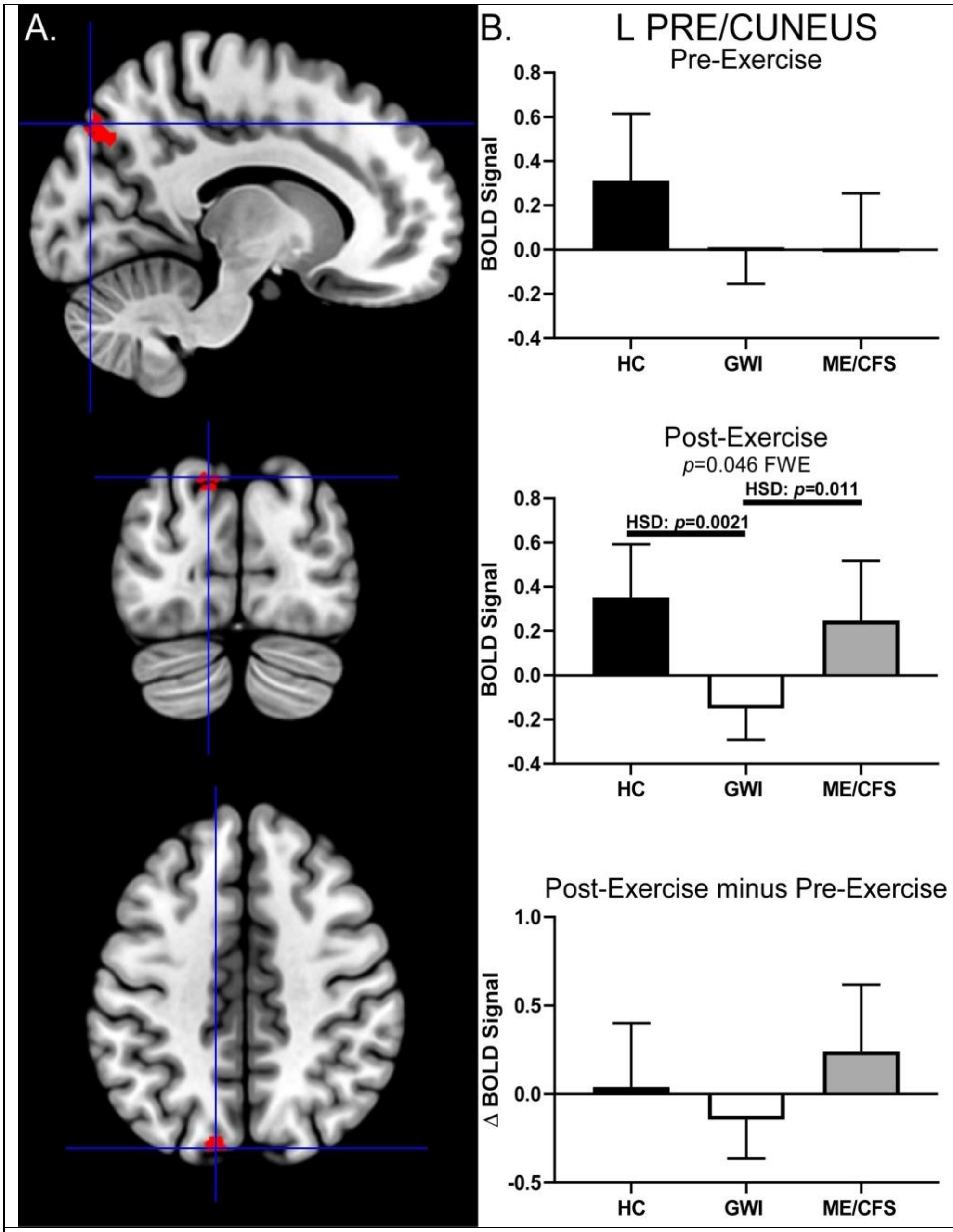

Supplementary Figure 2. Left cuneus/precuneus ROI identified by contrasting BOLD activity derived from the 2-Back>0-Back conditions in the HC, GWI, and ME/CFS groups via One-way ANOVA after exercise (cluster level: p=0.046, FWE; k_E=109). (A) Sagittal (top), coronal (middle), and transverse (bottom) slices of an MNI-standard brain, where crosshairs indicate the cluster's most active voxel (-12, -82, 44). (B) BOLD response for the 2-Back>0-Back condition (mean ± SEM) are shown for pre-exercise (top) and post-exercise (middle). DBOLD (bottom) is the post- minus pre-exercise BOLD response for the 2-Back>0-Back condition for the control (black bars), GWI (white bars), and ME/CFS (gray bars). Error bars represent 95% C.I.

Supplementary Figure 3.

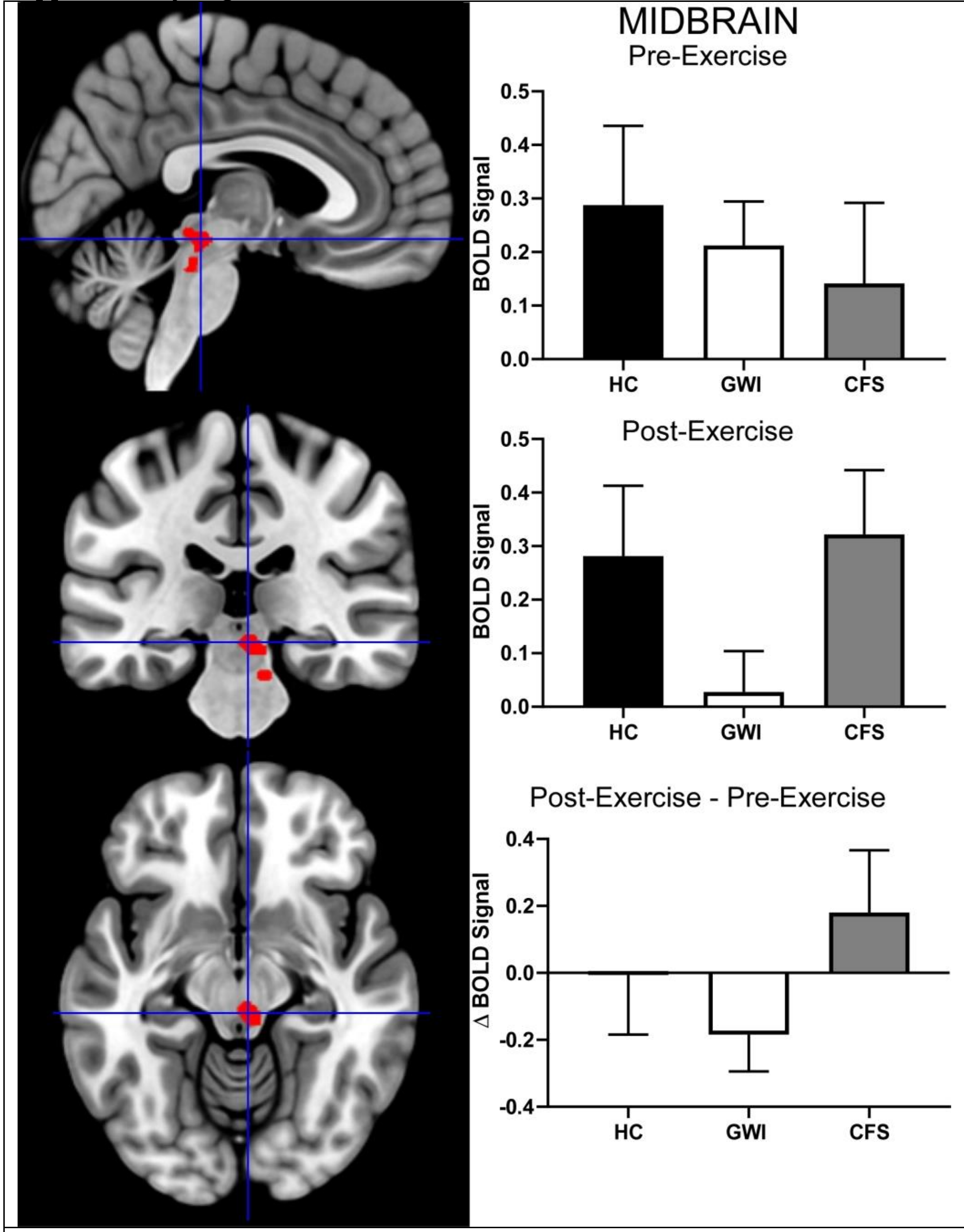

Supplementary Figure 3. Midbrain ROI identified by contrasting BOLD activity derived from the 2-Back>0-Back conditions in the HC, GWI, and ME/CFS groups via One-way ANOVA after exercise (cluster level: p=0.040, FWE; k_E=113). (A) Sagittal (top), coronal (middle), and transverse (bottom) slices of an MNI-standard brain, where crosshairs indicate the cluster's most active voxel (4, -28, -12). (B) BOLD response for the 2-Back>0-Back condition (mean ± SEM) are shown for pre-exercise (top) and post-exercise (middle). DBOLD (bottom) is the post- minus pre-exercise BOLD response for the 2-Back>0-Back condition for the control (black bars), GWI (white bars), and ME/CFS (gray bars). Error bars represent 95% C.I.

Supplementary Figure 4.

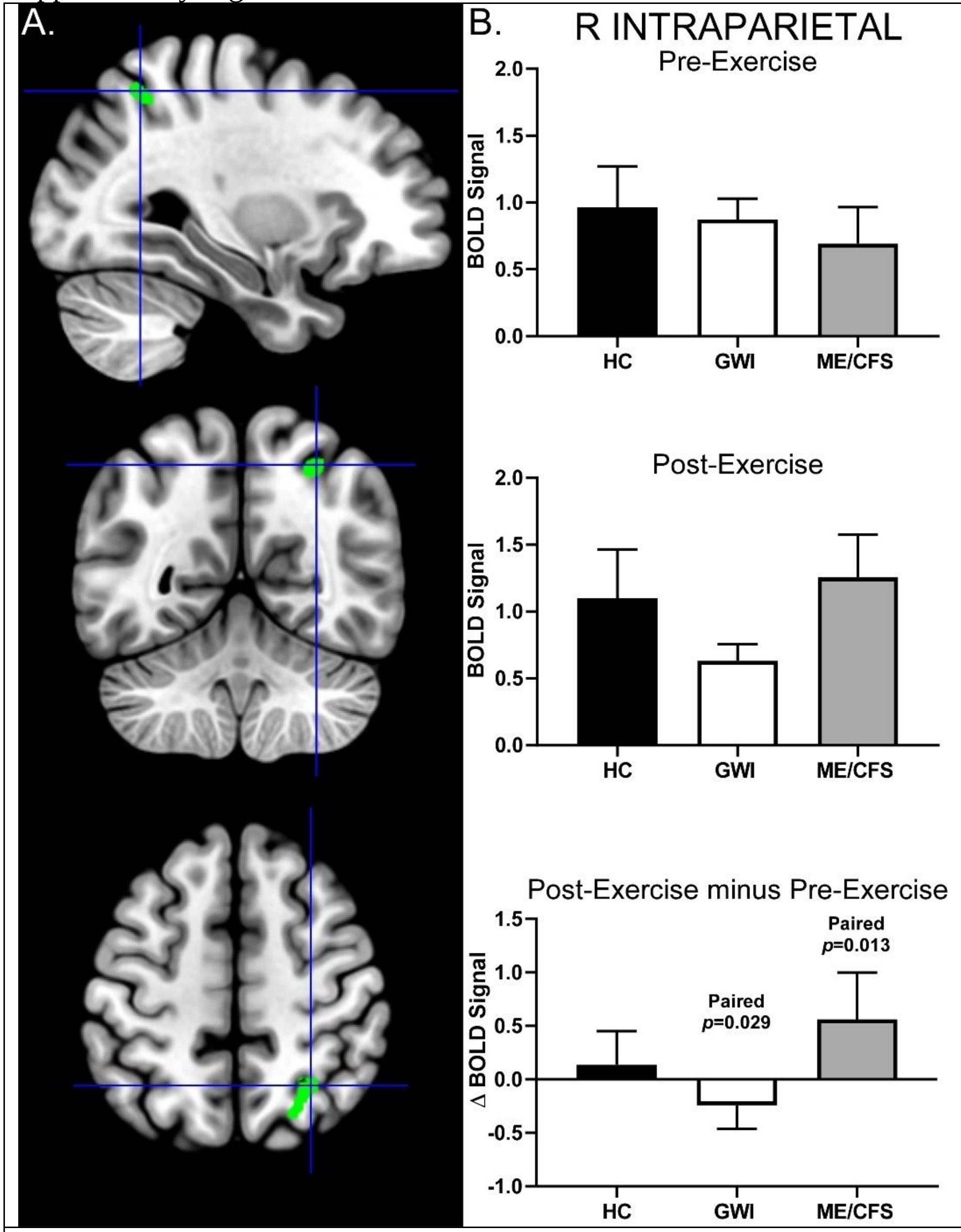

Supplementary Figure 4. Right intraparietal ROI identified by contrasting BOLD activity derived from the 2-Back>0-Back conditions in the HC, GWI, and ME/CFS groups via One-way ANOVA after exercise (cluster level: p=0.051, FWE; k_E=106). The ROI is green to signify that it is a trend and not statistically significant, like the ROIs in Supplementary Figures 1-3. (A) Sagittal (top), coronal (middle), and transverse (bottom) slices of an MNI-standard brain, where crosshairs indicate the cluster's most active voxel (30, -56, 52). (B) BOLD response for the 2-Back>0-Back condition (mean ± SEM) are shown for pre-exercise (top) and post-exercise (middle). DBOLD (bottom) is the post- minus pre-exercise BOLD response for the 2-Back>0-Back condition for the control (black bars), GWI (white bars), and ME/CFS (gray bars). Error bars represent 95% C.I.

Supplementary Figure 5

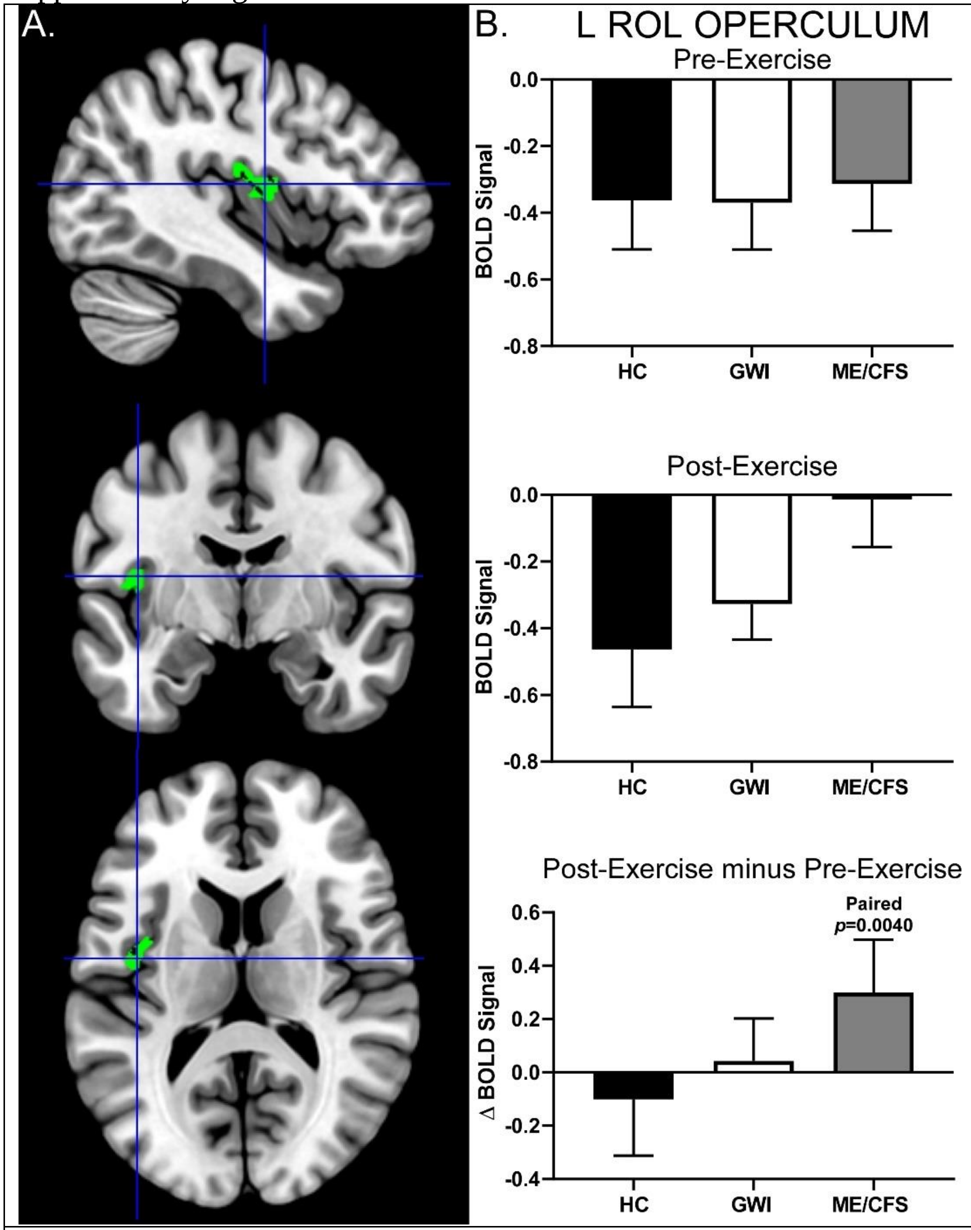

Supplementary Figure 5. Left Rolandic Operculum ROI identified by contrasting BOLD activity derived from the 2-Back>0-Back conditions in the HC, GWI, and ME/CFS groups via One-way ANOVA after exercise (cluster level: p=0.066, FWE; k_E=99). The ROI is green to signify that it is a trend and not statistically significant, like the ROIs in Supplementary Figures 1-3. (A) Sagittal (top), coronal (middle), and transverse (bottom) slices of an MNI-standard brain, where crosshairs indicate the cluster's most active voxel (4, -28, -12). (B) BOLD response for the 2-Back>0-Back condition (mean ± SEM) are shown for pre-exercise (top) and post-exercise (middle). DBOLD (bottom) is the post- minus pre-exercise BOLD response for the 2-Back>0-Back condition for the control (black bars), GWI (white bars), and ME/CFS (gray bars). Error bars represent 95% C.I.
